# Transformation networks of metal–organic cages controlled by chemical stimuli

**DOI:** 10.1039/d0cs00801j

**Published:** 2022-06-06

**Authors:** Elie Benchimol, Bao-Nguyen T. Nguyen, Tanya K. Ronson, Jonathan R. Nitschke

**Affiliations:** Yusuf Hamied Department of Chemistry, University of Cambridge Lensfield Road Cambridge CB2 1EW UK jrn34@cam.ac.uk

## Abstract

The flexibility of biomolecules enables them to adapt and transform as a result of signals received from the external environment, expressing different functions in different contexts. In similar fashion, coordination cages can undergo stimuli-triggered transformations owing to the dynamic nature of the metal–ligand bonds that hold them together. Different types of stimuli can trigger dynamic reconfiguration of these metal–organic assemblies, to switch on or off desired functionalities. Such adaptable systems are of interest for applications in switchable catalysis, selective molecular recognition or as transformable materials. This review highlights recent advances in the transformation of cages using chemical stimuli, providing a catalogue of reported strategies to transform cages and thus allow the creation of new architectures. Firstly we focus on strategies for transformation through the introduction of new cage components, which trigger reconstitution of the initial set of components. Secondly we summarize conversions triggered by external stimuli such as guests, concentration, solvent or pH, highlighting the adaptation processes that coordination cages can undergo. Finally, systems capable of responding to multiple stimuli are described. Such systems constitute composite chemical networks with the potential for more complex behaviour. We aim to offer new perspectives on how to design transformation networks, in order to shed light on signal-driven transformation processes that lead to the preparation of new functional metal–organic architectures.

## Abstract 2

This review highlights transformation networks created with coordination cages. Such synthetic stimuli-controlled networks can help elucidate biological signal transduction, as well as enabling new functions and applications.

## Graphical abstract

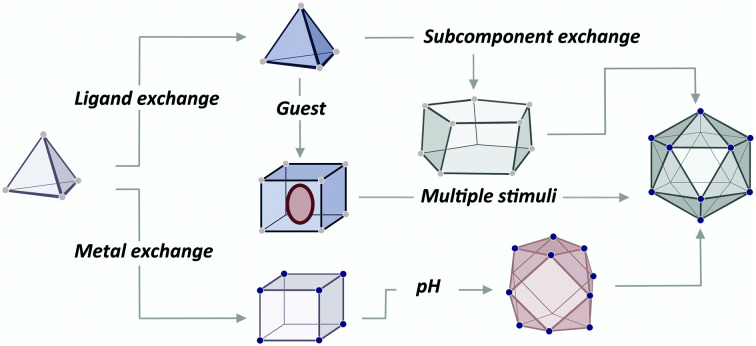

## 1. Introduction

Metal–organic cages^1–5^ are discrete three-dimensional (3D) structures comprising organic ligands and metal ions that self-assemble in solution. Their study has grown extensively over recent decades, driven by a desire to rationally design these self-assembled architectures in order to increase their structural^6–19^ and functional complexity.^20^ Many of these structures have well-defined internal pockets, within which the chemical reactivity and dynamics of guest molecules may be altered. Taking advantage of these inner cavities and their structural diversity, an increasing range of applications have been explored.^21^ Recent examples include the use of metal–organic cages for chemical separations,^22^ catalysis,^23,24^ luminescent sensing,^25,26^ as materials such as gels^25^ and for biomedical applications.^27–29^

An interesting feature of this class of compounds is the directional but dynamic nature of their metal–ligand bonds. Consequently, metal–organic cages can transform between geometrically-distinct structures formed from the same set of components, giving the cages an additional degree of flexibility. Such structures will have cavities that differ in their sizes and shapes and consequently may bind different guest molecules selectively. Structural transformations between cages thus offer the opportunity to alter their functions as well as their structure.

Metal–organic cages are sensitive to changes in their environment in the same way as biomolecular structures. Structural transformation is a well-known characteristic of proteins and other biomolecules.^30–33^ For example, enzymes can change their conformation to fit a target substrate through induced-fit processes. Mimicking biomolecules, metal–organic cages can dynamically reconfigure upon the application of stimuli to become more stable, or to switch on and off desired functionalities. Numerous stimuli have been employed to trigger these transformations, including the addition of new cage components, changes in stoichiometry, addition of guests, and changes in concentration, solvent and pH. Upon application of one of these stimuli, the components of a system can undergo rearrangement to reach a new thermodynamic mimimum, enabled by the dynamic nature of the coordination bonds.

Complementing direct coordination-driven self-assembly, the transformation of metal–organic structures using chemical stimuli provides alternative strategies to achieve structures of high complexity. In some instances, unprecedented structures have been obtained which were not accessible *via* direct metal–ligand self-assembly. The introduction of complementary building blocks is a straightforward strategy for obtaining thermodynamically favourable complexes, while the application of external stimuli can promote reversible transformations between structures within networks. However, a drawback of using chemical stimuli to transform cages is the possible buildup of by-products when additional compounds are added to the mixture.

Several excellent reviews have treated the stimuli-responsive transformations of supramolecular structures in general.^32,34^ Others have focused on more specific aspects such as light-triggered transformations,^35^ redox active assemblies,^36–38^ guest-induced reconfigurations^39^ and covalent post-assembly modification (PAM).^40,41^ In this review, we focus on chemically-controlled transformations of metal–organic cages and provide a library of recently reported strategies that transform cages and allow the creation of new architectures. Apart from touching on a few key precedents, we highlight work published over the past five years and thus not included in prior reviews.

In this review, we detail transformations between discrete architectures, where at least one of the species in the network is a three-dimensional metal–organic cage. Novel examples of transformations involving other types of self-assembled structures,^42^ including helicates,^43,44^ macrocycles,^45,46^ other one- and two-dimensional assemblies,^47,48^ and extended structures^49^ such as metal–organic frameworks,^50^ metallopolymers^51–53^ and soft materials,^54^ fall beyond the scope of this review. As others^41^ and our group^40^ have recently reviewed strategies to covalently modify coordination assemblies after their formation, we do not treat this type of chemical transformation herein. Finally, we also exclude redox responsive coordination cages, as recent developments in this field have been highlighted in comprehensive reviews from Sallé and co-workers.^37,38^

In order to clarify the key factors determining the outcome of cage transformation processes, we divide the review into sections based on the type of stimuli, which fall into two broad categories. Firstly, we highlight examples of architectures responsive to the introduction of competitive or complementary building blocks, which take the form of new ligands or metal ions, or even entire self-assembled species. Secondly, we summarise key examples of cage transformation triggered by external stimuli, such as the addition of templating guests, or changes in pH, solvent or concentration. Finally, we will highlight multi-stimuli responsive systems, where cages respond to several distinct stimuli to generate more complex chemical networks or to undergo structural transformations that cannot be triggered through exposure to a single stimulus.

To underline the utility of these transformation processes, we emphasise examples where the emergence of unprecedented architectures or new functions were observed. A greater understanding of the behaviour of these complex systems will enable the rational design of signal-driven transformation processes and contribute to the development of diverse fields, from systems chemistry to materials science.

## 2. Component-induced transformations

The addition of competitive building blocks to metal–organic cages can induce them to rearrange to form new, more stable structures. In some cases, the stoichiometry of the initial assembly is retained, while in other cases the addition of new components can change the metal-to-ligand ratio of the final structures. Transformations can take place *via* ligand exchange, metal exchange or subcomponent exchange for structures containing dynamic covalent bonds. Alternatively, entire self-assembled species can be added, leading to cage fusion processes whereby components from multiple structures are incorporated into new heteroleptic structures. In most cases, the systems incorporate the building blocks that form the most thermodynamically stable structures *via* self-sorting processes,^55^ which can be either integrative^56,57^ or narcissistic. In integrative processes, multiple building blocks are incorporated into a single structure, whereas in narcissistic processes, identical components generate homoleptic architectures.

### 2.1. Ligand-exchange-induced transformations

Metal–organic cages are able to transform between structures in the presence of competing ligands due to the lability of the metal–ligand bonds that hold them together. Weakly-binding ligands can be displaced by more strongly-binding ones, allowing for the formation of more thermodynamically stable structures. Transformations can take place with retention of stoichiometry if one ligand directly displaces another, or between structures of different stoichiometries, when competing ligands of different denticities are employed.

**Fig. 1:**
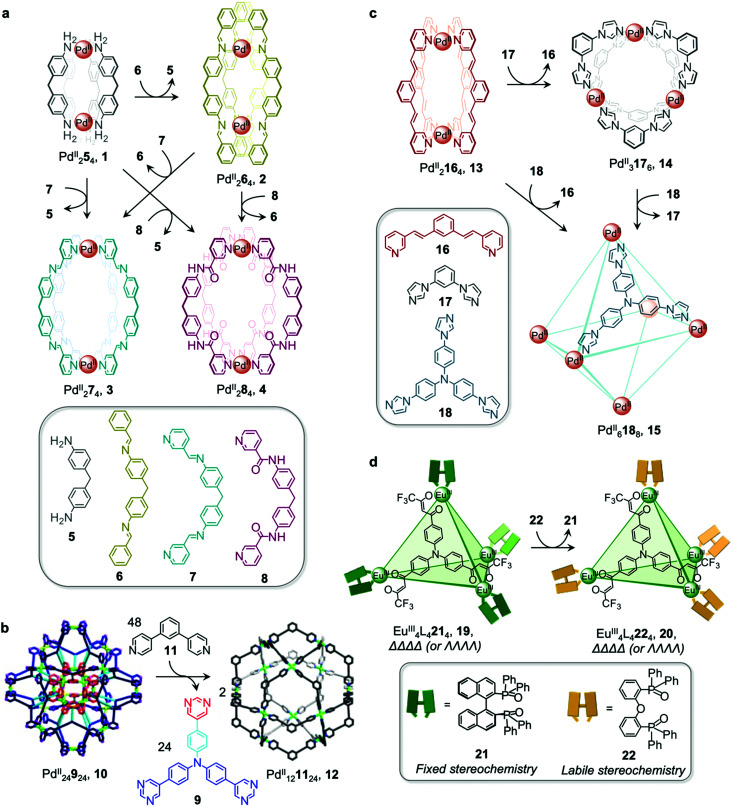
Examples of cage transformations triggered by ligand exchange. (a) A network of interconverting Pd^II^_2_L_4_ cages driven by the binding hierarchy of the ligands to the Pd^II^ centres.^58^ (b) Formation of cage 12 from the double-layered ‘pregnant molecular nanoball’ cage 10.^60^ Adapted from ref. 60 with permission from American Chemical Society, copyright 2021. (c) Transformation between Mukherjee's cages 13–15, attributed to enthalpic factors.^61^ (d) Chiral memory observed upon exchange of the stereochemically fixed ancillary ligand 21 with the more labile 22 to transform cage 19 to 20.^62^

Chand and co-workers reported a network composed of four different Pd^II^_2_L_4_ cages 1–4, which interconvert *via* ligand exchange pathways (Fig. 1a).^58^ The transformations are driven by the difference in strength of the Pd–N bonds, in the following order: amine–Pd < imine–Pd < pyridine–Pd. The introduction of four equivalents of ligand 6, 7 or 8 to a solution of cage 1 results in the release of ligand 5 together with the formation of cage 2, 3 or 4, respectively. Similarly, ligand 6 is released when cage 2 is combined with ligand 7 or 8, giving rise to cage 3 or 4. However, mixing cage 4 with ligand 7 or cage 3 with ligand 8 does not result in complete ligand substitution, forming a mixture of cages instead. The results suggest that there is no hierarchical preference between ligand 7 and 8. The binding affinity order of the ligands to Pd^II^ is therefore 5 < 6 < 7 ≈ 8.

The ligand exchange reactions employed in this system enable the cavity size of the Pd^II^_2_L_4_ cages to either be retained or expanded in a controlled manner. Conversion from cage 1 to cage 2, 3 or 4 also occurs following covalent modification of the free amine residues of 5.

Mukherjee *et al.* also took advantage of differences in ligand strength to transform double-layered Pd^II^_24_9_24_ cage 10 into hollow spherical Pd^II^_12_11_24_ cage 12, which was first reported by the Fujita group.^59^ This transformation occurs following introduction of 48 equivalents of competitive bis-pyridine ligand 11, leading to the release of 24 equivalents of tris-pyrimidine ligand 9 and a change in the stoichiometry of the complex (Fig. 1b).^60^ Compared to tris-pyrimidine ligand 9, the bis-pyridine ligand 11 is a better donor, thus allowing for the formation of stronger Pd–N bonds in the resulting cage 12. In addition to being enthalpically driven, the transformation process is also inferred to be driven by entropic factors, as two equivalents of cage 12 are formed from a single equivalent of cage 10.

The Mukherjee group subsequently employed a similar strategy to create a transformation network between three Pd^II^ cages, 13–15 (Fig. 1c). When treated separately with Pd^II^(NO_3_)_2_, bis-pyridyl ligand 16, bis-imidazole ligand 17 and tris-imidazole ligand 18 form Pd^II^_2_L_4_ lantern-shaped cage 13, Pd^II^_3_L_6_ barrel 14 and Pd^II^_6_L_8_ sphere 15, respectively. When ligands 17 and 18 are added separately to a solution of cage 13, the more strongly-coordinating imidazole ligands displace the pyridyl ligand 16, resulting in the formation of cages 14 and 15 respectively.^61^ Competition experiments between imidazolyl ligands 17 and 18 yielded cage 15 as the thermodynamic product following reaction with Pd^II^(NO_3_)_2_ in a 6 : 4 : 3 ratio. The preferential formation of 15 is inferred to be due to a guest templation effect from six encapsulated NO_3_^−^ anions, overcoming any entropic preference for the smaller cage 14. When a mixture of the three ligands 16–18 is allowed to react with enough Pd^II^ for only one cage to form, the exclusive formation of cage 15 is observed.

Ligand exchange can also be used to preserve chiral information within cages. This approach was illustrated by Yan *et al.*, who prepared enantiopure lanthanide cage 20 from precursor cage 19 (Fig. 1d).^62^ Cage 20 is racemic if constructed through direct metal–ligand self-assembly, but *ΛΛΛΛ*-20 and *ΔΔΔΔ*-20 can be formed stereoselectively through displacement of the stereochemically-fixed ancillary ligand *R*- or *S*-bis(diphenylphosphoryl)-1,1′-binaphthyl (*R*/*S*-BINAPO) 21 with the stereochemically labile bis[2-(diphenylphosphino)phenyl]ether oxide (DPEPO) 22, as a result of retention of the stereochemistry of the cage framework during the cage-to-cage transformation.

The initial diastereoselective synthesis of Eu^III^_4_L_4_(*R*/*S*-BINAPO)_4_ tetrahedral cage 19 is controlled by the sterically bulky chiral *R/S*-BINAPO ancillary ligand and mechanical coupling through the rigid tritopic ligands. Introduction of excess DPEPO to a solution of cage 19 results in complete substitution of the BINAPO ligand with retention of the stereochemical information imparted by ligand 21.

The transformation from cage 19 to 20 is concentration- and temperature-dependent, indicating that it can happen *via* an associative or dissociative pathway. In dilute solution or at higher temperatures, the degree of dissociation of the *R/S*-BINAPO ancillary ligands increases, leading to loss of the stereochemical information imparted by these ligands. In contrast, higher concentrations and lower temperatures allow the chiral BINAPO units to stay incorporated until their displacement, enabling retention of helical handedness at the metal centres. In the associative pathway, a single Eu^III^ metal centre with multiple binding sites is inferred to increase its coordination number so that it can bind to both the BINAPO and DPEPO at the same time. The final enantiopure cage 20 is therefore composed only of achiral components. In addition to retaining the chirality of the original cage framework, 20 also retains the circularly polarized luminescence (CPL) properties of 19, which arise from its Eu^III^ metal centres. A luminescence dissymmetry factor (*g*_lum_) of 0.11 was measured for 20, representing about half of the value for the initial enantiopure cage 19. Both cages also display luminescent quantum yields of up to 81% and 68% for 19 and 20, respectively. A similar stereochemical memory phenomenon was observed by our group, in a process occurring *via* subcomponent exchange on a Fe^II^_4_L_4_ cage as discussed in Section 2.2 below.^63^

**Fig. 2:**
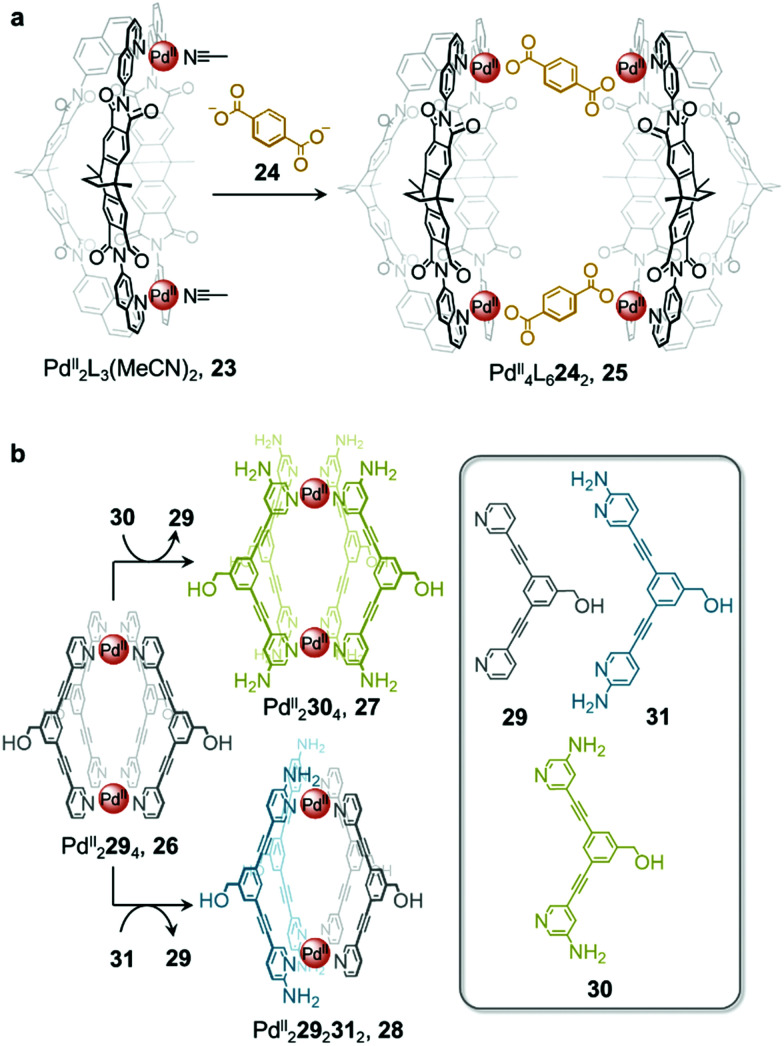
(a) Heteroleptic cage 25 assembled *via* dimerization of two equivalents of cage 23 upon addition of ligand 24.^64^ (b) Transformation of cage 26 to homoleptic 27 and heteroleptic 28*via* ligand displacement involving a more electron-rich ligand.^65^

In addition to enabling transformations between homoleptic cages, ligand exchange processes have also been demonstrated to provide a useful pathway for the formation of heteroleptic assemblies. Clever and co-workers reported a pill-shaped dimeric Pd^II^_4_L_6_24_2_ cage 25, assembled from dimerization of two Pd^II^_2_L_3_(MeCN)_2_ cages 23 upon reaction with a benzene-1,4-dicarboxylate ligand 24 (Fig. 2a).^64^ The carboxylate ligands displace bound acetonitrile from the Pd^II^ centres, bridging the two bowl-shaped complexes 23 and resulting in the formation of cage 25. With a larger inner cavity, cage 25 is able to encapsulate two C_60_ or C_70_ fullerenes, as compared to cage 23, which only binds a single fullerene.

Selective ligand exchange reactions are not only governed by ligand shape and coordination vectors, but are also influenced by ligand functionality. The Crowley group have demonstrated how electron-rich substituents in proximity to ligand binding sites influence the outcome of self-assembly through both electronic and steric effects. Unsubstituted Pd^II^_2_L_4_, cage 26 transforms into either homoleptic Pd^II^_2_L_4_ cage 27, or heteroleptic cage, 28, upon the addition of electron-rich amino-substituted ligands 30 and 31 (Fig. 2b).^65^*meta*-Substituted ligand 30 is a stronger donor than 29 and is thus able to rapidly displace the weaker ligand from the parent cage 26 to form the homoleptic cage 27.

Despite being an even stronger donor than ligand 30, ligand 31, with amino substituents *ortho* to the coordinating nitrogen, generates the metastable heteroleptic *cis*-Pd^II^_2_29_2_31_2_ cage 28. This cage is stabilised by hydrogen-bonding interactions between the adjacent 2-amino units of the *cis*-coordinated ligands. Although the homoleptic Pd^II^_2_31_4_ cage was predicted to be the ultimate thermodynamic product of the system, the *ortho*-amino substituents of ligand 31 were inferred to prevent further ligand substitution after heteroleptic *cis*-Pd^II^_2_29_2_31_2_, cage 28 has formed. Steric clashes and lone-pair repulsions with incoming 31 ligands were thus inferred to create a kinetic barrier to further ligand displacement within 28.

Heteroleptic cage 28 can only be formed cleanly *via* ligand displacement, with mixtures of cages obtained from the direct combination of ligands 29 and 31 with [Pd^II^(MeCN)_4_](BF_4_)_2_ in a 1 : 1 : 1 ratio. This observation suggests that preorganisation of the initial Pd^II^_2_L_4_ cage 26 is crucial to the clean formation of 28. This study thus demonstrates the power of cage-to-cage transformations to yield heteroleptic assemblies that are difficult to access by other pathways.

### 2.2. Transformations induced through subcomponent exchange

Cages composed of ligands bearing dynamic covalent bonds, which form *in situ* by subcomponent self-assembly, have grown in interest over the last decade.^66–73^ These systems enable cage-to-cage transformations to occur *via* exchange of aldehyde or amine subcomponents, rather than complete ligands. Such processes are driven by the formation of more thermodynamically stable complexes when new subcomponents are introduced, driven by the difference in electronic and steric properties of various subcomponents, or the chelate effect. Subcomponent exchange can enable the exterior of cages to be functionalized,^74^ stereochemical information to be transferred,^63^ or the spin state of metal ions to be modified.^75^

In the simplest case, electron poor anilines at the periphery of a cage are displaced by electron rich ones, as exemplified by early work from our group,^74^ and more recent work from Gu and co-workers^76^ using a series of enantiopure Fe^II^_4_L_6_ cages constructed from chiral amines. More complex networks of transformations between diverse structures that incorporate a single subcomponent backbone have also been realized, as illustrated by a transformation network reported by us in 2013, consisting of multiple Cd^II^_2_L_3_ triple helicates, Cd^II^_3_L_3_ triangular circular helicates, Cd^II^_4_L_4_ tetrahedral cages, and a Cd^II^_12_L_18_ hexagonal prism, all sharing a common 4,4′-diformyl-3,3′-bipyridine building block.^77^ Transformations between network members take place upon the introduction of more nucleophilic amines, which trigger imine exchange due to the more electron-rich character of the added amine or chelate effects.^77^

**Fig. 3:**
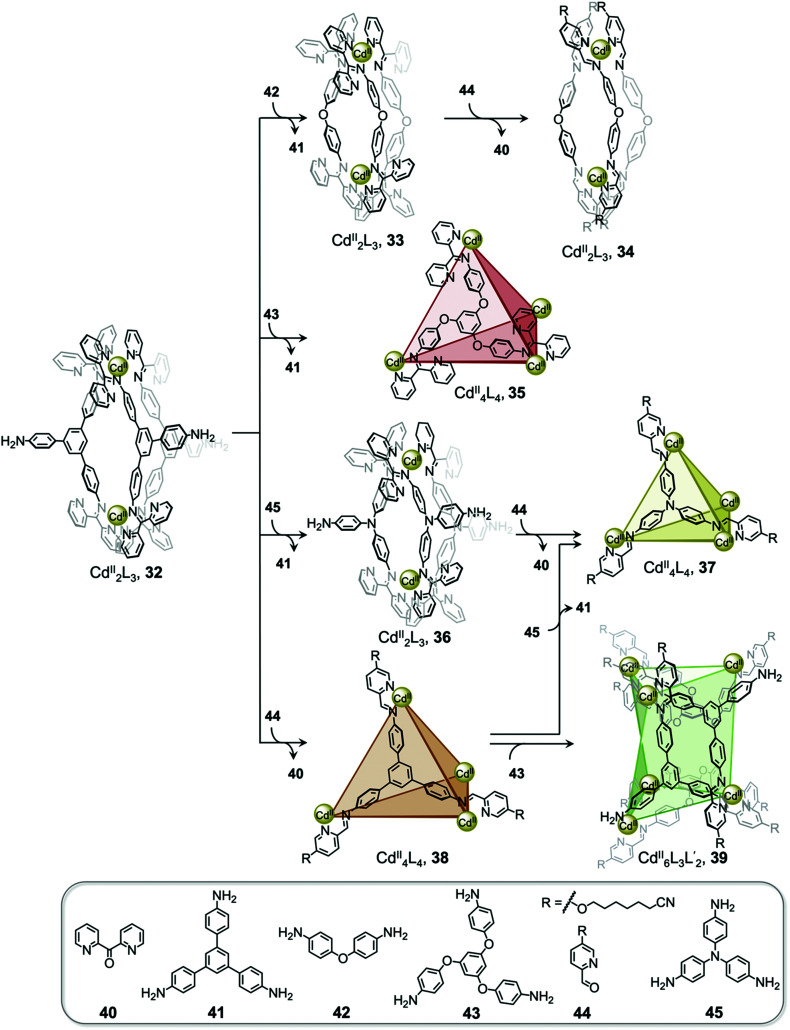
Network of interconverting structures 32–39, with transformations driven by electronic effects and relief of steric hindrance.^78^

Interconversion between structures not only results in the formation of more thermodynamically stable structures but can also generate complexes with new properties, such as guest selectivity, allowing specific functions to be switched on or off upon transformation. Recently, we reported a network of interconverting structures 32–39, driven by subcomponent exchange processes (Fig. 3).^78^ The network illustrates the transformation of one Cd^II^_2_L_3_ helicate into another, helicates into Cd^II^_4_L_4_ tetrahedra, interconversion between different tetrahedral structures, and finally formation of heteroleptic Cd^II^_6_L_6_L′_2_ trigonal prism 39.

Two distinct types of transformation were employed in this system, starting from Cd^II^_2_L_3_ helicate 32. Firstly, the central trianiline 41 is displaced when 32 reacts with the more nucleophilic anilines 42 and 45, to form helicates 33 and 36. Similarly, the more electron-rich triamine 43 replaces the less electron-rich 41, transforming helicate 32 to tetrahedron 35, and converting tetrahedron 38 to heteroleptic prism 39.

The transformation forming tetrahedron 35 is also driven by bound triflate anions acting as templates, and may be favoured entropically as more free particles are present in solution following the substitution reaction. Introducing the more nucleophilic aniline 45 to tetrahedron 38 fosters the transformation to tetrahedron 37 and the release of the less nucleophilic aniline 41.

Secondly, di(2-pyridyl)ketone 40, which builds steric hindrance into complexes, can be displaced by 2-formylpyridine 44, in a reaction driven by release of steric encumbrance around the metal centres after conversion. As a result, more stable helicate 34 is formed from the less stable 33. Similarly, the addition of 44 and additional Cd^II^ to the helicates 32 and 36 drives formation of tetrahedra 38 and 37, respectively, accompanied by release of di(2-pyridyl)ketone 40 in both cases.

The transformations between the structures of Fig. 3 led to changes in their host–guest properties, thus allowing different guests to be encapsulated by different network members. For example, the initial helicate 32 does not encapsulate guests, but converts to tetrahedron 35, which binds triflate anions, and to tetrahedron 38, which binds cyclohexane. Transformation thus allows one of these guests to be selectively taken up from solution. The tetrahedron 37 and trigonal prism 39 are also able to bind anionic guests, such as AsF_6_^−^ and SbF_6_^−^.

**Fig. 4:**
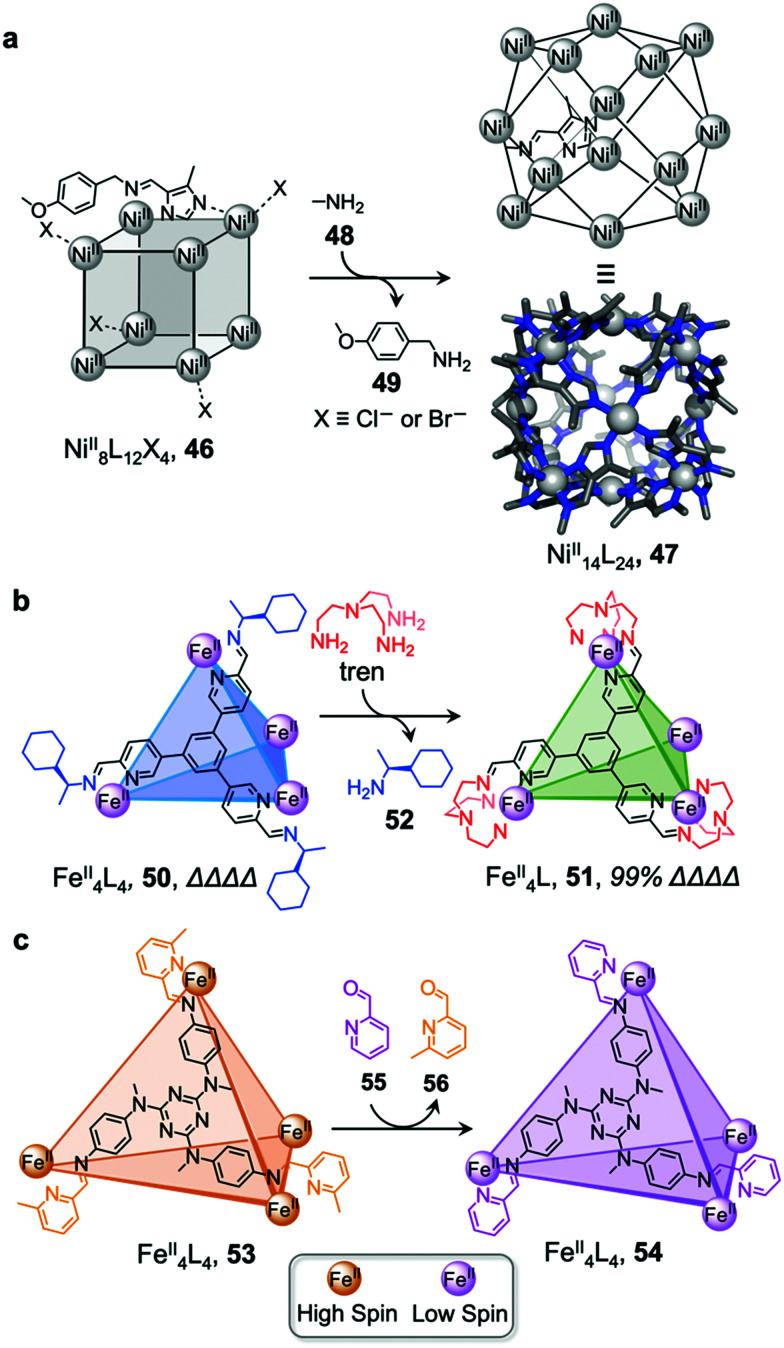
Examples of cage transformation triggered by subcomponent exchange. (a) Exchange of bulky 4-methoxybenzylamine by methylamine leads to the transformation of cage 46 into cage 47.^79^ (b) An enantiopure *ΔΔΔΔ* cage 51 is formed by exchange of a chiral amine by achiral tren through a stereochemically retentive pathway.^63^ (c) Aldehyde exchange transforms high-spin 53 into low-spin 54, with spin-state switching a result of the release of steric crowding around the iron(ii) metal centres.^75^

Li and co-workers demonstrated the transformation of a Ni^II^_8_L_12_X_4_ (X = Cl^−^ or Br^−^) cubic structure 46 into a rhombic dodecahedral Ni^II^_14_L_24_ cage 47, by subcomponent exchange of 4-methoxybenzylamine 49 for methylamine 48 (Fig. 4a).^79^ The steric bulk of the 4-methoxybenzylamine was inferred to be an essential factor for stabilising the tetrahedral Ni^II^ centres in cubic structure 46. When the less bulky methylamine subcomponent replaces 4-methoxybenzylamine, the tetrahedral Ni^II^ centres become unstable, leading some to adopt a square planar geometry and triggering transformation to the more complex yet more stable cage 47.

Subcomponent exchange can conserve or alter the stereochemistry of cages. We reported a homochiral *ΔΔΔΔ*-Fe^II^_4_L_4_ cage 50 assembled from (*S*)-1-cyclohexylethylamine 52 and a rigid trialdehyde subunit (Fig. 4b).^63^ Exchange of the chiral amine for achiral chelating tris(2-aminoethyl)amine (tren) leads to the formation of enantiopure *ΔΔΔΔ*-cage 51 or a racemic mixture, through either a stepwise, stereochemically retentive or a dissociative pathway. Depending on the concentration and the presence of free Fe^II^ ions, the parent Fe^II^_4_L_4_ cage framework can remain intact or dissociate. At low concentration, the transformation process happens *via* the dissociative pathway, resulting in the loss of chiral information. Higher concentrations favour the retentive pathway, which conserves stereochemical information. The presence of free Fe^II^ also drives the formation of the enantiopure structure, by coordinating to excess tren and preventing initial demetallation of the cage.

In other cases, the spin properties of coordination cages are altered through cage transformation. We showed that aldehyde exchange can drive the transformation of high-spin cage 53 to low-spin cage 54 (Fig. 4c).^75^ The change in spin state was inferred to be a consequence of reduced steric hindrance around the metal centres. The coordination environments of the Fe^II^ centres in high spin 53, incorporating methyl-substituted subcomponent 2-formyl-6-methylpyridine 56, experience steric hindrance and exhibit high-spin properties. Substituting 56 residues with the less hindered subcomponent 2-formylpyridine 55, results in conversion of the high-spin Fe^II^ centres to a low-spin configuration. The cage-to-cage transformation also modulates the cage stability towards electron-rich 4-methoxyaniline, allowing selective cage disassembly and guest release.

### 2.3. Metal ion induced transformations

The structures of metal–organic cages are dependent on the interplay between the type and arrangement of ligand binding sites and the preferred coordination geometries of metal ions. Cage structures can thus be controlled in some cases through modification of stoichiometry or by the introduction of metal ions with different coordination preferences. In the first strategy, additional equivalents of the metal ion already present in the structure are added, leading to the formation of a new structure with a different metal/ligand stoichiometry. In these examples, the product of the transformation process incorporates the metal ions from the original structure. In order to employ this strategy, the original structure must contain unused coordination sites. In the second strategy, a different and more strongly coordinating metal ion is introduced. In contrast to the first strategy, the newly added metal ions outcompete the existing ones, thus forming new structures. Since the original metal ion is fully or partially displaced, the original structure does not need to be coordinately unsaturated. This section will discuss examples of both strategies.

**Fig. 5:**
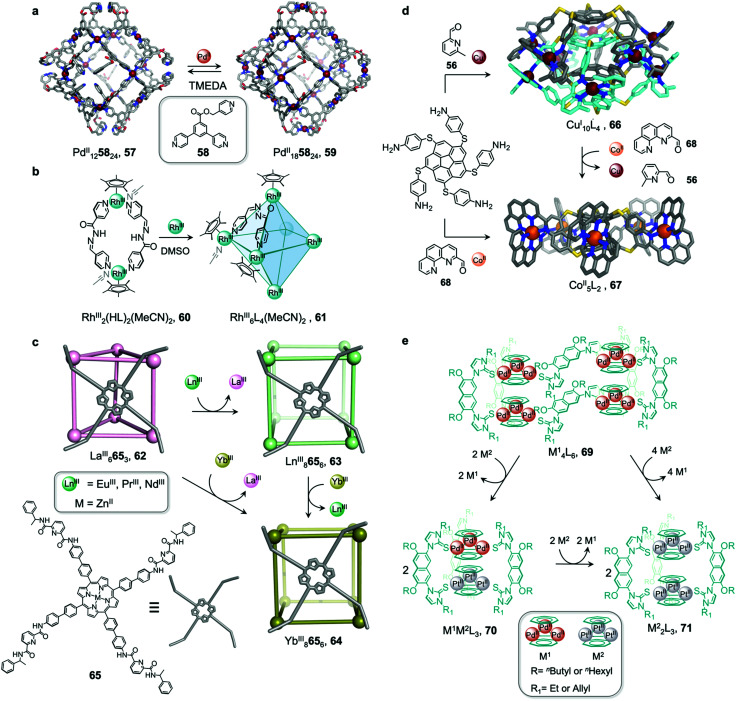
Examples of metal-ion induced cage-to-cage transformations. (a) Addition of a Pd^II^ salt to cage 58 promoted coordination of its free pyridyl arms to the Pd^II^ centres, thus forming stellated cuboctahedron 59 with enclosed faces.^80^ (b) Addition of Rh^III^ transforms macrocycle 60 into cage 61.^81^ (c) Transmetallation allows the formation of a series of Ln^III^_8_L_6_ (Ln^III^ = Pr^III^, Nd^III^ or Eu^III^) cubes, 63 and Yb^III^_8_L_6_ cube 64, which could not be formed *via* direct metal–ligand assembly.^82^ (d) Two Co^II^_5_L_2_ cages 67 were formed from Cu^I^_10_L_4_ cage 66*via* displacement of the Cu^I^ ions and 2-formyl-6-methylpyridine by Co^II^ ions and 2-formylphenanthroline.^84^ (e) The replacement of tripalladium (Tr_2_Pd^II^_3_) by triplatinum (Tr_2_Pt^II^_3_) clusters drove the conversion of 69 to the intermediate (Tr_2_Pd^II^_3_)(Tr_2_Pt^II^_3_)L_3_70 and final triple helicate (Tr_2_Pt^II^_3_)_2_L_3_ cage 71.^85^

In an example of the first strategy, Fujita *et al.* reported the formation of a stellated cuboctahedron 59 from precursor cage 57 (Fig. 5a) *via* modification of the stoichiometry.^80^ When tris(pyridyl) ligand 58 was mixed with [Pd^II^(MeCN)_4_](BF_4_)_2_ in a 2 : 1 ratio, cuboctahedral Pd^II^_12_L_24_ cage 57 self-assembled selectively due to the high stability of its Pd^II^_12_L_24_ core. Two pyridyl moieties from each ligand coordinate to the Pd^II^ centres, leaving the third one uncoordinated. Following addition of further Pd^II^, the free pyridyl arms bind to the Pd^II^ centres, closing the open faces and affording Pd^II^_18_L_24_ stellated cuboctahedron 59. The conversion of 57 to 59 not only increases the degree of complexity of the overall architecture, but also influences the degree of surface enclosure of the cage and may thus influence the host–guest properties of the cage. The process can be reversed following addition of *N,N,N*′*,N*′-tetramethylethylenediamine (TMEDA), which removes metal ions from the stellated faces and thus regenerates cage 57. This process offers a potential gate opening–closing mechanism, which might be used to trap large guests inside 59.

Similarly, Jin and co-workers reported the conversion of Rh^III^_2_HL_2_(MeCN)_2_ macrocycle 60 to octahedral Rh^III^_6_L_4_(MeCN)_2_ cage 61, supported by half-sandwich {Cp*Rh^III^} (Cp* = η^5^-pentamethylcyclopentadienyl) metal centres (Fig. 5b).^81^ Due to their flexible design, the 4-pyridinecarbaldehyde isonicotinoyl hydrazine ligand can act as either a ditopic ligand, through its two pyridyl donors, or a tritopic ligand when it deprotonates and adopts a bent arrangement, exposing an anionic NO-chelating binding site that enables it to coordinate to three different Rh^III^ vertices. The meta-stable macrocycle thus readily converts into cage 61 upon the addition of a source of {Cp*Rh^III^} in DMSO.

In the second strategy, architectures are transformed through addition of a metal ion with a different preferred coordination geometry. Such metal exchange processes have allowed the formation of complexes that could not be obtained *via* direct metal–ligand self-assembly routes. Transformations involving the addition of a metal ion with a similar coordination geometry but different size or coordination strength can result either in conservation of the original framework, or may lead to more dramatic structural transformations. The addition of a metal ion with a different preferred coordination geometry, in contrast, can only trigger transformation to a new structural framework, if a clean transformation occurs.

Sun *et al.* reported a near infrared (NIR) emitting Yb^III^_8_L_6_ cube 64, which could only be prepared using a transmetallation strategy (Fig. 5c). Self-assembly of enantiopure porphyrin-based tetrakis-tridentate ligand 65 with La^III^(OTf)_3_ yields coordinatively-unsaturated La^III^_6_L_3_ triangular prism 62, while reaction with other Ln^III^ salts yield a series of Ln^III^_8_L_6_ (Ln^III^ = Pr^III^, Nd^III^ or Eu^III^) cubes 63.^82^ However, the direct reaction of ligand 65 with Yb^III^(OTf)_3_ does not result in the formation of the expected Yb^III^_8_L_6_ cube 64 (Fig. 5c). The authors inferred that the high formation constant for this complex hinders the error correction process required to form the most thermodynamically stable complex from kinetically trapped intermediates. Instead, post-assembly metal exchange of cage 62 with Yb^III^(OTf)_3_ allows its transformation into Yb^III^_8_L_6_64. Cage 64 can also be obtained *via* the same metal-ion metathesis strategy from lanthanide-based cube 63. A cascade transformation from trigonal prism 62 to Eu^III^_8_L_6_ cube 63 and then 64 was also demonstrated. It was hypothesized that slight differences in the ionic radii and coordination strength between the lanthanides combined with release of the torsional strain of the ligand were the driving forces for these successive transformations. Owing to their larger cavity and more optimal arrangement of porphyrin panels for stacking with guests, the Ln^III^_8_L_6_ cubes exhibit selective binding of polycyclic aromatic hydrocarbon guests, while La^III^_6_L_3_ prism 62 does not bind these guests. Thus, the transformation from 62 to 64 triggers uptake of coronene guests from solution.

A similar transmetallation strategy, involving displacement of a weak-binding, labile metal ion for a stronger-binding, more inert one was used by Han *et al.* to transform a triply interlocked Ag^I^ cage to its Au^I^ analogue without altering the intertwined framework of the catenated cages.^83^

In another study by our group, an *S*_10_-symmetric catenated Cu^I^_10_L_4_ cage 66 transforms into two smaller discrete *D*_5_-symmetric Co^II^_5_L_2_ cages 67*via* a combined metal and subcomponent exchange process (Fig. 5d).^84^ Cage 66 forms from two five-fold interlocked Cu^I^_5_L_2_ cages, and is stabilised by van der Waals interactions between stacked corannulene moieties in the interlocked structure. The addition of 2-formylphenanthroline 68, and Co^II^ to 66 leads to the *in situ* formation of tridentate ligand sites suitable for octahedral coordination with the newly introduced Co^II^ centres, resulting in displacement of the Cu^I^ ions and the subcomponent 2-formyl-6-methylpyridine, 56. The cage-to-cage transformation from 66 to 67 is both enthalpically driven, by the stronger coordination of octahedral Co^II^ compared to tetrahedral Cu^I^, and entropically favoured, by an increase in the number of discrete species in solution.

In some cases, metal exchange can occur in stepwise fashion at the vertices of structures, resulting in the formation of an intermediate containing multiple metal ions. Han *et al.* reported a structural transformation process driven by metal-cluster exchange (Fig. 5e).^85^ A tube-like organometallic (Tr_2_Pd^II^_3_)_4_L_6_, cage 69 was constructed from bifunctional sulfur ligands coordinated to cycloheptatrienyl (Tr) trimetallic palladium clusters (Tr_2_Pd^II^_3_). Taking advantage of the difference in Pd–S and Pt–S binding strengths, the replacement of tripalladium (Tr_2_Pd^II^_3_) by triplatinum (Tr_2_Pt^II^_3_) clusters can occur without any disruption of the metal-metal bonding in the clusters. Introducing a Pt-cluster Tr_2_Pt^II^_3_ to a solution of cage 69 results in the formation of a triple helicate (Tr_2_Pt^II^_3_)_2_L_3_ cage 71. During the process, the intermediate (Tr_2_Pd^II^_3_)(Tr_2_Pt^II^_3_)L_3_70 incorporating trimetallic sandwich complexes of both Pd^II^ and Pt^II^ clusters was detected. The authors inferred the large difference in the structures of 69 and 71, despite the apparently similar coordination preferences of the two metal ions, resulted from subtle differences in the structures of the trimetallic clusters, and the M–S bond distances.

### 2.4. Transformation through cage fusion

Cage fusion, where additional components are added in the form of complete assembled structures, is another strategy to transform cages and obtain unpreceded structures. During the process, the parent cages dissociate, and their building blocks reassemble into more stable heteroleptic structures that carry features inherited from the parent cages. Fujita described the first example of cage fusion, forming heteroleptic triply interlocked Pd^II^ and Pt^II^ cages that were more stable than the homoleptic cages prepared from their two constituent pyridyl-based ligands, laying the groundwork for obtaining mixed-ligand structures *via* cage fusion strategies.^86^ The generality of this approach was further demonstrated by Mukherjee's preparation of related interlocked Pt^II^ or Pd^II^ cages, employing an imidazole-containing ligand in place of one of the pyridyl-based ligands.^87^ Another key approach was developed by Stang, using a mixture of carboxylate and pyridyl ligands to form a series of heteroleptic Pt^II^-based architectures.^39^

**Fig. 6:**
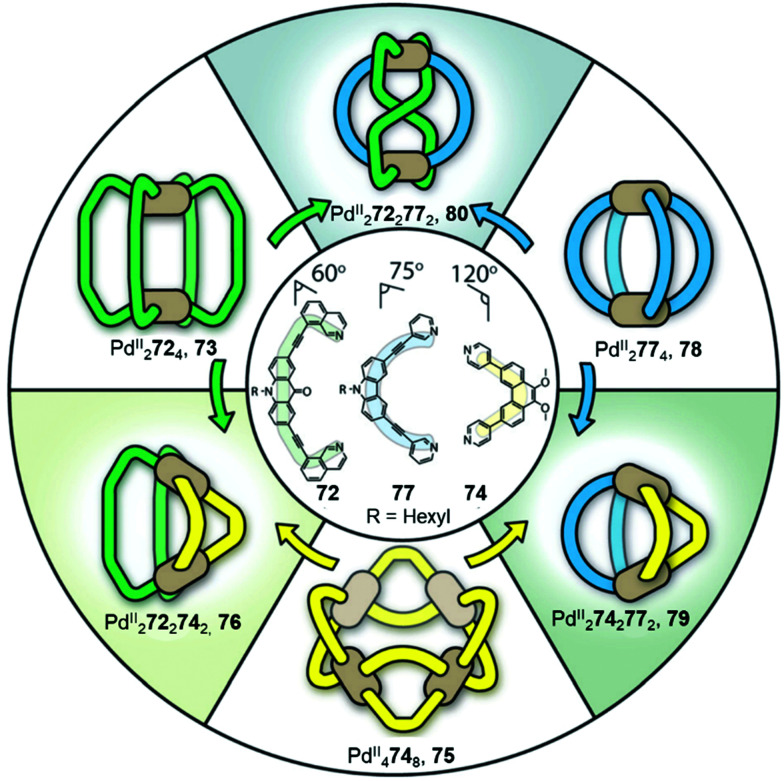
Heteroleptic cages 76, 79 and 80 formed *via* cage fusion of the corresponding homoleptic cages and subsequent integrative self-sorting of ligands. Thermodynamically stable heteroleptic 76 is favoured when cages 79 and 80 are mixed in the presence of catalytic Cl^−^, due to complementarity between the ligand binding angles.^88^ Adapted from ref. 88 with permission from John Wiley and Sons, copyright 2021.

The value of the cage fusion strategy for forming structures of high complexity has also been elegantly demonstrated by the Clever group, who developed a strategy to favour heteroleptic cages based on the geometric complementarity of carefully designed ligands (Fig. 6). Mixing homoleptic cages Pd^II^_2_72_4_, 73 and Pd^II^_4_74_8_, 75 in a 2 : 1 ratio results in the formation of a more thermodynamically stable *cis*-Pd^II^_2_72_2_74_2_ heteroleptic cage 76, *via* an integrative self-sorting process, based on geometric complementarity between the binding angles of the two ligands.^88^

With a distinctly bent cavity, cage 76 preferably encapsulates 2,7-naphthalene disulfonate over the 2,6-substituted isomer. Owing to its bent molecular shape, the encapsulated 2,7-naphthalene disulfonate can interact with the Pd^II^ centres of the cage and position itself between the acridone backbones of the ligands, stabilised by aromatic stacking interactions. In contrast, the linear guest 2,6-naphthalene disulfonate was observed to bind less strongly to cage 76, as the geometry of the guest did not allow this substrate to fit as well into the bent pocket of the cage.

An extension of this study examined the structural rearrangement of multiple homoleptic and heteroleptic cages.^56,57^ Heating a mixture of cages Pd^II^_4_74_8_, 75 and Pd^II^_2_77_4_, 78 forms another heteroleptic Pd^II^_2_74_2_77_2_ cage, 79 while the heteroleptic Pd^II^_2_72_2_77_2_ cage, 80 forms as the major product from the reaction between Pd^II^_2_72_4_, 73 and Pd^II^_2_77_4_, 78. Cage 80 bears a unique ‘doubly bridged figure-of-eight’ topology, in which ligands 72 are highly twisted, adopting an *anti*-configuration, in contrast to the *syn*-configuration observed in all of the homoleptic cages, and resulting in *trans*-coordination of the two isoquinoline donors at the Pd^II^ centres.

The study also highlights the ability of heteroleptic architectures to interconvert through ligand exchange and structure re-organisation. For instance, cages 79 and 80 convert into cage 76 upon the introduction of ligand 72 or 74, respectively. Cage 76 is the thermodynamic product of both transformations as a result of having the best match between ligand bite angles. In contrast, mixing all three heteroleptic cages in a 1 : 1 : 1 ratio leads to the formation of kinetically favourable cage 80 as the major product, which then partially converts to the more thermodynamically stable cage 76 following the introduction of catalytic Cl^−^, which acts as a competing ligand to aid lability.

**Fig. 7:**
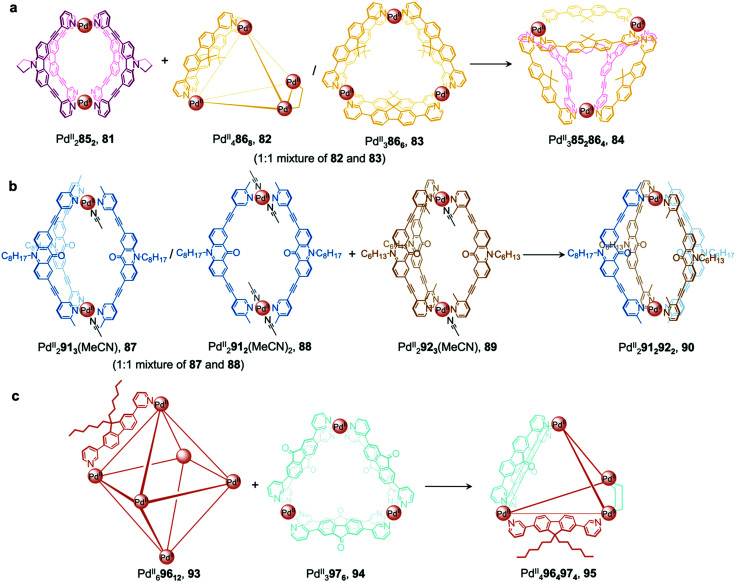
Examples of cage-to-cage transformations occurring *via* cage fusion reported by the Clever group. (a) Reaction of homoleptic dinuclear Pd^II^_2_85_2_ cage 81 with a mixture of Pd^II^_3_86_6_ and Pd^II^_4_86_8_ cages 82 and 83 led to the formation of heteroleptic *pseudo*-tetrahedron 84.^11^ (b) The selective formation of heteroleptic Pd^II^_2_91_2_92_2_ cage 90 is dictated by the steric hindrance of the *ortho* and *para* methyl substituents on the pyridyl rings of ligands 92 and 91, positioned inside and outside with respect to the cage cavity.^89^ (c) Unprecedented heteroleptic Pd^II^_4_96_4_97_4_ cage 95 formed from mixing Pd^II^_6_96_12_ cage 93 with Pd^II^_3_97_6_ triangular ring 94.^90^

More recently, Clever *et al.* extended their shape complementarity approach to prepare a more complex heteroleptic pseudo-tetrahedron 84, incorporating a new ligand 85, which consists of two ligand 77 subunits joined by a flexible covalent backbone (Fig. 7a). Combination of dinuclear homoleptic cage Pd^II^_2_85_2_, 81 with the mixture of a Pd^II^_4_86_8_ tetrahedron 82 and a Pd^II^_3_86_6_ trimeric ring 83 led to the formation of pseudo-tetrahedron 84.

A related cage-to-cage transformation strategy, this time employing steric crowding, selectively forms heteroleptic Pd^II^_2_91_2_92_2_ cage 90 when homoleptic precursor [Pd^II^_2_92_3_(MeCN)] reacts with a 1 : 1 mixture of [Pd^II^_2_91_3_(MeCN)]/[Pd^II^_2_91_2_(MeCN)_2_] (Fig. 7b).^89^ Ligands 92 and 91 bear methyl substituents on their pyridyl rings, positioned either *ortho* or *para* to the ligand backbone, respectively, thereby fixing their position inside or outside the cage with respect to the cavity. Both substituent positions produce steric hindrance, preventing the formation of coordinatively saturated homoleptic Pd^II^_2_L_4_ cages. Combination of the unsaturated precursors led to the formation of the more thermodynamically stable heteroleptic *cis*-Pd^II^_2_91_2_92_2_ cage 90, where a mixture of the two different ligand types allows two interior and two exterior methyl substituents to be accommodated at each vertex without steric strain.

More recently, a heteroleptic Pd^II^_4_96_4_97_4_ cage 95, was reported by Clever, contributing to the diverse library of cages formed from cage fusion strategies (Fig. 7c).^90^ Combination of a Pd^II^ salt with bent fluorenone-based ligand 97 in a 1 : 2 ratio formed Pd^II^_3_97_6_ triangular ring 94 as the major product alongside other Pd^II^_*n*_L_2*n*_ assemblies. In contrast, reaction of a bulkier analogue, ligand 96 with Pd^II^ in the same ratio formed larger Pd^II^_6_96_12_ cage 93 as the sole product, as this structure is able to accommodate the sterically demanding ligands without steric clashes. Mixing cages 93 and 94 such that there is an equimolar amount of each ligand allowed for the formation of heteroleptic Pd^II^_4_96_4_97_4_ pseudo-tetrahedral structure 95*via* an integrative self-sorting process. This structure incorporates the less bulky 97 along the two edges bridged by two ligands, leaving the bulky 96 to occupy the four remaining singly-bridged edges, thus avoiding the steric strain that would be incurred if two bulky ligands occupied the same edge.

The guest-binding properties of cage 95 are different to its precursor cages. Whilst triangular ring 94 and octahedron 93 are able to encapsulate up to one and three bis-sulfonate guests respectively, cage 95 encapsulates two guests. Furthermore, the emission of ligand 97 is retained when cage 95 forms, in contrast to many other cases where Pd^II^-coordination causes luminescence quenching.

**Fig. 8:**
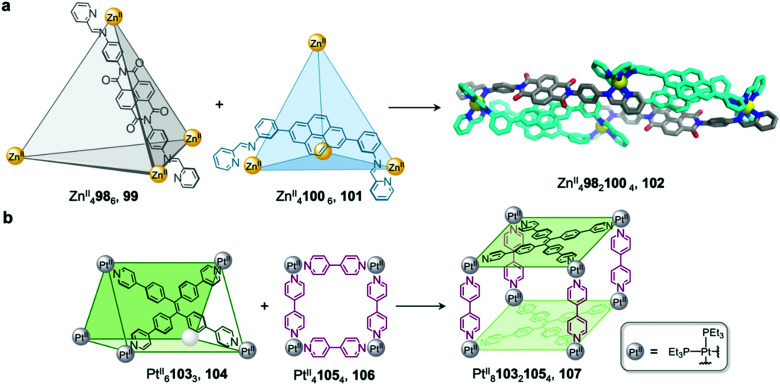
Examples of cage-to-cage transformations occurring *via* cage fusion. (a) Our triple-decker cage 102 formed through fusion of two Zn^II^_4_L_6_ tetrahedral cages 99 and 101.^92^ (b) Yan's heteroleptic cage 107 formed through the fusion of trigonal prism 104 and macrocycle 106.^93^

We demonstrated that cage fusion can occur between structures with similar as well as different geometries.^91^ Two Zn^II^_4_L_6_ tetrahedral cages, 99 and 101, assemble from pyrene and naphthalenediimide (NDI) building blocks with similar sizes but different geometries (Fig. 8a). When mixed together in a 2 : 1 ratio, the two cages reassembled into a triple-decker heteroleptic Zn^II^_4_98_2_100_4_ sandwich-like structure 102. This structure exhibits extensive aromatic stacking interactions between the ligand backbones, which are arranged into two pyrene–pyrene–NDI stacks. Despite the unusual donor-donor-acceptor stacking, this new cage was found to be thermodynamically stable, representing an example of complete integrative self-sorting between cages *via* a fusion process.

Similarly, architectures with different shapes or sizes can also recombine to form new heteroleptic structures.^92^ Yan *et al.* have reported a heteroleptic Pt^II^_8_103_2_105_4_ cage 107 that forms through fusion between Pt^II^_6_103_3_ trigonal prism 104 and Pt^II^_4_105_4_ macrocycle 106 (Fig. 8b).^93^ Trigonal prism 104 exhibits strong fluorescence due to aggregation-induced emission of the tetraphenylethylene (TPE) ligand, which is rigidified upon cage formation. Upon transformation into cage 107, the fluorescence of the TPE panels is red-shifted and partially quenched *via* photoinduced electron transfer, giving rise to a new method to track the cage transformation.

**Fig. 9:**
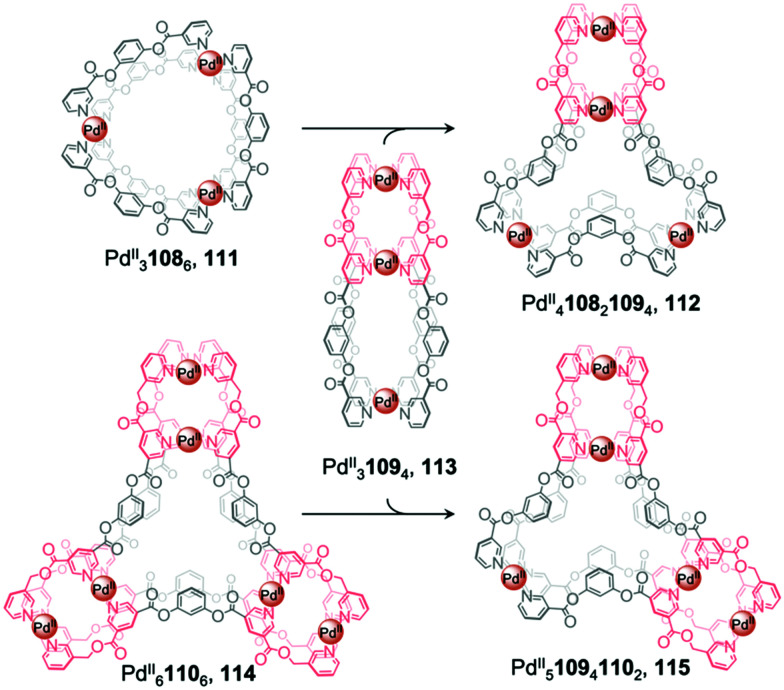
Chand's multi-cavity cages Pd^II^_4_108_2_109_4_, 112 and Pd^II^_5_109_4_110_2_, 115, formed through fusion of cage 113 with either cage 111 or 114, respectively.^94^

Recently, Chand *et al.* reported multi-cavity heteroleptic cages Pd^II^_4_108_2_109_4_, 112 and Pd^II^_5_109_4_110_2_, 115 constructed *via* the fusion of other multi-cavity cages (Fig. 9).^94^ Ditopic ligand 108 with two terminal pyridyl donors adopts a bent conformation and forms homoleptic Pd^II^_3_L_6_ cage 111. Ligands 109 and 110, with three and four pyridyl donors respectively, generate homoleptic Pd^II^_3_109_4_ cage 113 and Pd^II^_6_110_6_ cage 114, respectively. Cage 113 can be visualised as a linear combination of two distinct [Pd_2_L_4_] units, while 114 resembles a [Pd_3_L_6_] core surrounded by three [Pd_2_L_4_] units. Mixing cage 113 with either 111 or 114 results in the formation of the heteroleptic cages 112 and 115, respectively, which also contain a central [Pd_3_L_6_] pocket but this time attached to one or two [Pd_2_L_4_] termini. The driving force for cage fusion was inferred to be the formation of the favourable [Pd_3_L_6_] subunit from the flexible ester linked fragments resembling 108, which are present within the longer ligands.

The favourability of forming structures bearing the [Pd_3_L_6_] moiety was illustrated by cage assembly *via* ligand self-sorting pathways. Mixing ligands 108 and 109 with Pd^II^(NO_3_)_2_ led to the formation of heteroleptic cage 112 instead of homoleptic cages 111 and 113. Similarly, the integrative self-sorting of ligands 109 and 110 occurred during reaction with Pd^II^, forming cage 115. In addition, the homoleptic and heteroleptic cages were observed to interconvert *via* multiple ligand exchange pathways. Introduction of a stoichiometric quantity of competing ligand results in the consumption of the original cage followed by the formation of a new cage. Addition of ligand 109 or 110 to cage 111 results in the formation of cages 112, 114 or 115. Similarly, introduction of ligand 110 to cages 112 or 113 triggers the displacement of ligands 108 and 109 respectively, forming cage 115 in both cases.

The conjoined cages selectively encapsulate different guests within their multiple pockets. The smaller [Pd_2_L_4_] pockets selectively encapsulate small anionic guests, such as NO_3_^−^ and halides, which also act as templates for the structures, whilst only the DMSO solvent is encapsulated in the central [Pd_3_L_6_] moieties.

## 3. Transformations induced by external stimuli

Whereas the previous section describes how the building blocks of coordination cages can be modified or exchanged to transform architectures, this section focuses on how various external stimuli can be employed for the same purpose. Guest templates, concentration, and solvent may also impact the most stable structure expressed by a given set of building blocks. The presence or absence of these stimuli can lead to a rearrangement of the structural elements already present in a system to form a new thermodynamically favoured structure, allowing systems to adapt to their environment. Understanding stimuli-responsive cage-to-cage transformations is also crucial for the design of cage-based functional materials.

### 3.1. Guest induced transformations

Metal–organic cages exhibit the ability to encapsulate one or multiple guest molecules within their inner pockets.^95^ Cavity design has been a key point of focus in the construction of coordination cages, with most cage-based applications^24,66^ arising from their binding properties. Cages can encapsulate cargoes as diverse as anions,^96^ gases,^97^ fullerenes,^98^ dyes,^99^ natural products^100^ and drug molecules.^101,102^ Studies have shown that guest recognition is dictated by intermolecular interactions between host and guest, as well as their size and shape complementarity. In some cases, cages are able to adapt their cavities to accommodate guests through expansion or contraction of flexible cavities,^103–106^ while in other cases guests can template the formation of an entirely new host with more favourable binding properties for the guest, as we discuss below.

Raymond and co-workers paved the way for investigations into this kind of transformation^107^ in their pioneering study of the guest-induced interconversion between a helicate and a tetrahedral Ga^III^ cage triggered by the addition of NMe_4_^+^ cations which bind inside the cavity of the anionic cage. In contrast to Raymond's anionic cages,^108^ a majority of coordination cages are positively charged owing to their cationic metal vertices, thus many of them accommodate anionic species favourably within their cavities.^95,109^ Depending on their sizes and shapes, anionic guests can therefore drive cage-to-cage transformations assisted by induced-fit phenomena.^110^

**Fig. 10:**
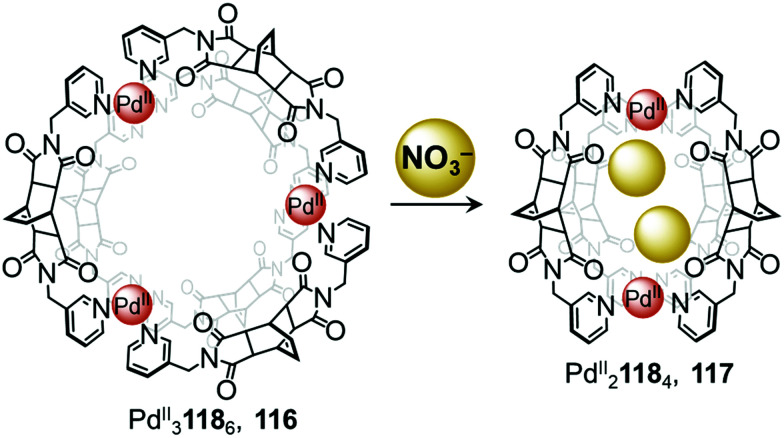
Anion driven conversion between ring 116 and cage 117. Nitrate anions act as templates, driving the formation of the smaller Pd^II^_2_118_4_ cage.^111^

In 2018, Su and co-workers observed conversion between a ring-like Pd^II^_3_L_6_ structure and a lantern-shaped Pd^II^_2_L_4_ cage induced by anion metathesis (Fig. 10).^111^ Mixing ligand 118 with [Pd^II^(MeCN)_4_](BF_4_)_2_ affords the Pd^II^_3_118_6_ cage 116, where the three Pd^II^ centres are arranged in a triangular configuration. Treatment with the smaller anion NO_3_^−^ converts the assembly into the Pd^II^_2_118_4_ cage 117 by means of a stronger induced-fit phenomenon.

In a recent report, Jung and co-workers highlighted another anion driven transformation.^112^ They initially prepared a Pd^II^_3_X_6_L_2_ trigonal prism by mixing a *C*_3_-symmetric ligand with K_2_PdX_4_ (X = Cl^−^ and Br^−^). The corresponding Pd^II^_3_I_6_L_2_ prism can also be obtained by irradiating Pd^II^_3_Cl_6_L_2_ in the presence of additional CH_2_I_2_. Moreover, addition of Ag^I^BF_4_ and two extra equivalents of ligand transforms the initial Pd^II^_3_X_6_L_2_ (X = Cl^−^ and Br^−^) prisms into a Pd^II^_6_L_8_ cube. This conversion is reversible when an excess of NH_4_Cl or ^*n*^Bu_4_NBr is introduced, regenerating the trigonal prismatic architecture.

Anions can also drive the interlocking of coordination cages. Kuroda^113^ and Clever^114–116^ have reported several ground-breaking studies on doubly or triply interpenetrated structures resulting from anion binding. Drawing inspiration from these early studies, the Clever group have recently expanded the scope of their interlocked assemblies.

**Fig. 11:**
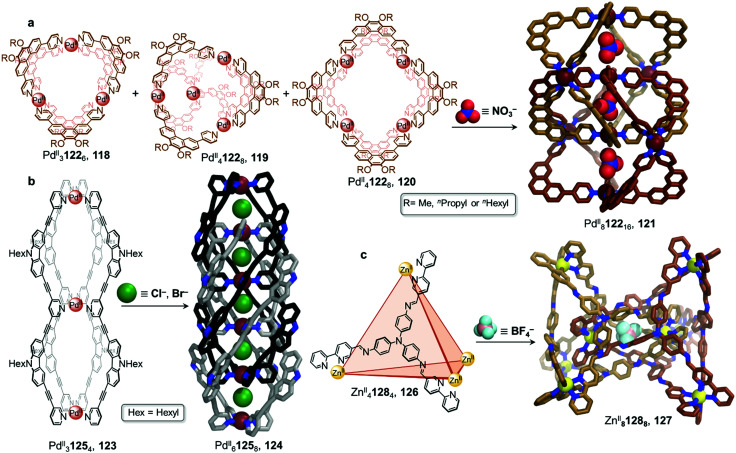
Examples of anion-driven transformations producing interlocked cages. (a) Anion templation allows a mixture of assemblies to be driven towards a single interlocked Pd^II^_8_L_16_ cage 121. Alkyl chains in the X-ray structure of 121 are omitted for clarity.^117^ (b) The double-cage 123 was transformed into a highly interpenetrated architecture by stoichiometric addition of chloride, affording new species 124 with five consecutive cavities. Alkyl chains in the X-ray structure of 124 are omitted for clarity.^118^ (c) Interpenetrated metal–organic cage 127 is formed *via* subcomponent self-assembly and templation by ClO_4_^−^ or BF_4_^−^ anions. The use of labile Zn^II^ plays a crucial role in allowing dynamic reconfiguration of the components. Only the centrally bound BF_4_^−^ anion is shown in the structure of 127.^119^

In 2018, the group presented a novel Pd^II^_8_122_16_ giant “Hopf link” catenane 121 (Fig. 11).^117^ Mixing phenanthrene-spaced ligand 122, which possesses a 60° bite angle, with [Pd^II^(MeCN)_4_](BF_4_)_2_ yields a mixture of assemblies 118–120. However, in the presence of NO_3_^−^ and after heating at 70 °C for 24 h, 121 forms quantitatively. X-ray structure analysis unambiguously confirmed the *D*_2d_-symmetric Pd^II^_8_122_16_ structure and indicated that it was comprised of two interlocked *D*_4h_-symmetric Pd^II^_4_L_8_ cages 120, creating three distinct cavities where NO_3_^−^ anions were accommodated. Once again, the size of the anion drives the cage-to-cage transformation and facilitates cage interpenetration.

The Clever group then reported the catenation of an even more complex structure, leading to the formation of a Pd^II^_6_125_8_ cage 124 with five consecutive cavities (Fig. 11b).^118^ Non-interlocked Pd^II^_3_125_4_ cage 123 was first obtained by mixing the ligand 125 with [Pd^II^(MeCN)_4_](BF_4_)_2_ in acetonitrile for 6 h at 70 °C. The peanut-like assembly, comparable to two conjoined Pd^II^_2_L_4_ cages, has two physically segregated cavities. Two catenation scenarios could be envisaged for 123. The first would lead to a polycatenane where neighbouring cages 123 would be interlocked with each other by means of a single cavity only, generating an infinite chain. The second scenario, which was observed upon Cl^−^ or Br^−^ addition, led to a multi-interpenetrated dimer 124, creating five cavities where the halide guests were bound. These newly formed dimers were also found to aggregate into larger colloidal discs with a diameter of 12 to 16 nm.

In another system, Gan and co-workers reported the dimerization of a lantern-shaped cage based on an amide-linked dipyridyl ligand.^120^ While a monomeric Pd^II^_2_L_4_ cage is the kinetic product, longer reaction times result in conversion to a Pd^II^_4_L_8_ interlocked structure. The authors suggested that in addition to the templating BF_4_^−^ anion, this transformation was favoured by aromatic stacking between ligands in the catenated cage as well as hydrogen bonding involving the amide moieties.

In a similar manner to the aforementioned examples, catenation of a tetrahedral cage was carried out in a subcomponent self-assembled system by the Duan group (Fig. 11c).^119^ Tetrahedron 126 was isolated by combination of tris(4-aminophenyl)amine and 2,2′-bipyridine-5-carbaldehyde subcomponents, forming ligand 128, and Zn^II^(OTf)_2_. Upon further addition of ClO_4_^−^ to 126, the authors observed the formation of triply-interlocked Zn^II^_8_L_8_ catenane 127, consisting of two tetrahedral cages interlocked *via* one vertex of each cage, such that a vertex of one cage resides in the centre of the other. The loss of symmetry of the final architecture was indicated by splitting of the ^1^H NMR signals. Although addition of BF_4_^−^ gave partial conversion as well, this phenomenon was not observed in the presence of PF_6_^−^, demonstrating a strong induced-fit process between the host and the guest.

The crystal structure of the BF_4_^−^ salt of 127 revealed that the large inner cavities of the tetrahedral cages were divided into seven individual parts in 127, each of which was occupied by a BF_4_^−^ anion in the solid state. The inner pocket in the centre of the structure was inferred *via* titration experiments to be most important for the anion templated formation of 127. Kinetic studies confirmed a second order reaction in relation to the concentration of tetrahedron 126. Further control experiments revealed that the catenation process did not proceed with more inert metal centres, such as Fe^II^ or Co^II^, which form stronger M^II^–N bonds and are thus less dynamic. Br^−^ and I^−^ also templated the formation of 127, allowing the catenation process to be reversed through addition of Ag^I^.

**Fig. 12:**
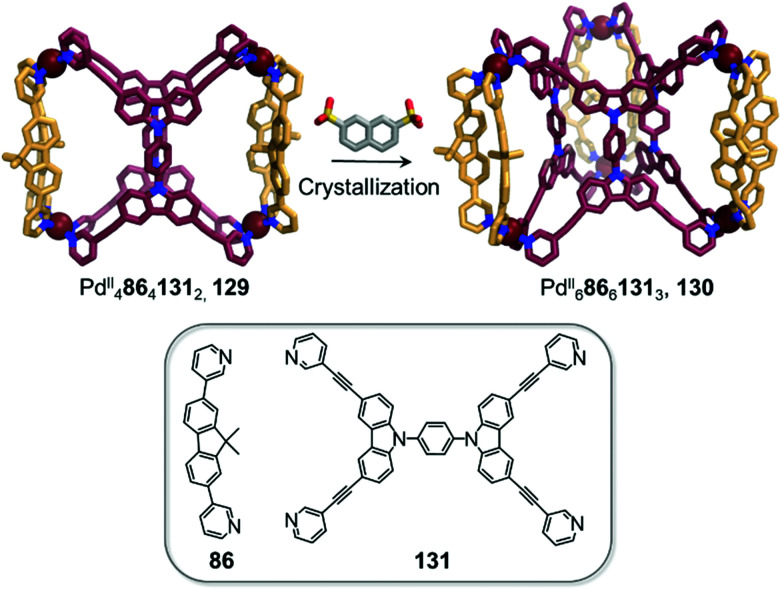
Cage-to-cage transformation through crystallisation in the presence of 2,7-naphthalene disulfonate anions. Anions do not play a templating role but act instead as bridges between the Pd^II^ centres in the crystal lattice.^88^

Clever and co-workers highlighted another phenomenon that can occur in the presence of anionic guests (Fig. 12).^11^ In an extension of their shape-complementarity strategy,^88^ combination of ditopic 86, tetratopic 131 (consisting of two ditopic ligands bridged by a rigid aromatic backbone) and Pd^II^ in a 1 : 2 : 2 ratio yields a new Pd^II^_4_86_4_131_2_ cage 129, as confirmed by X-ray structure analysis. The authors chose 2,7-naphthalene disulfonate as a guest for 129, reasoning that its size, shape and ability to form hydrogen bonds would render it a good fit for the two outer cavities of the assembly, as subsequently confirmed by NMR titrations. Surprisingly, single crystal analysis revealed the presence of an unprecedented Pd^II^_6_86_6_131_3_ architecture 130, where the disulfonates were not encapsulated inside the cavities in the solid state. In contrast, they bridged distinct 130 cages *via* C–H⋯O–S hydrogen bonds as well as sulfonate–Pd^II^ interactions.

**Fig. 13:**
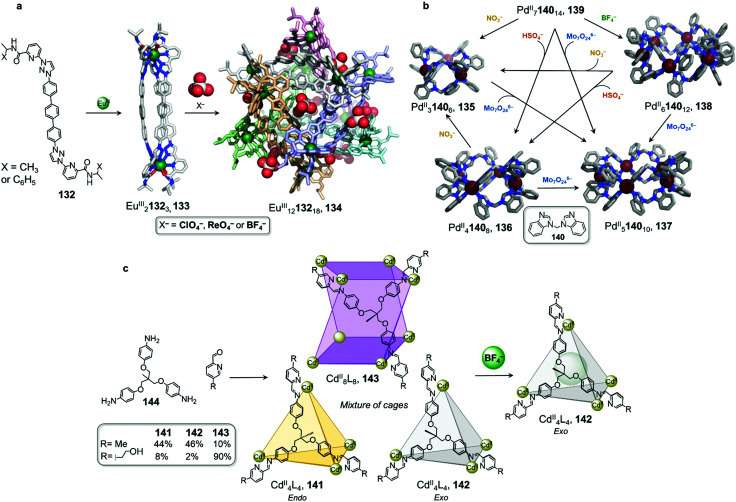
Examples of anion-driven cage-to-cage transformations. (a) Anion-induced formation of supramolecular helicate hexamer 134. At very high concentrations, helicate 133 aggregates into superstructure 134 where anions play the role of templates, holding the six building blocks together *via* hydrogen-bonding interactions.^121^ (b) Transformation network of cages, where all transformations are driven by anion metathesis. The induced-fit phenomenon drives reconfiguration of the assemblies towards the most stable host–guest complex 137.^123^ (c) A library of cages obtained by subcomponent self-assembly collapsed to produce uniquely species 142 following introduction of BF_4_^−^.^124^

Sun and coworkers have recently reported a transformation where Eu^III^_2_L_3_ helicates aggregated into a tertiary-like structure upon anion templation (Fig. 13a).^121^ They first utilised *C*_2_-symmetric ligand 132 with tridentate binding sites in combination with Eu^III^(OTf)_3_ to form Eu^III^_2_132_3_ triple helicate 133. Surprisingly, replacing the triflate anions by perchlorate yields another species that predominates at higher concentrations.

DOSY NMR indicated the formation of a single species much larger than the previously-isolated helicate. Further ESI-TOF-MS analysis allowed the authors to confirm the formation of a large Eu^III^_12_132_18_ architecture 134. The crystal structure confirmed the formation of a (Eu^III^_2_132_3_)_6_ hexamer, where the helicates 133 stack in an intertwined manner to form a supramolecular assembly reminiscent of protein tertiary structures such as the insulin hexamer.

Anions play an important role in the templation of the assembly *via* the formation of hydrogen-bonding interactions with the polarised triazole protons of the ligand. Multiple ligand-ligand aromatic stacking interactions also contribute to the overall stability of the cage.

Hexamer 134 was observed to form by substitution of the triflate anions by perchlorate in a pre-formed solution of the Eu^III^_2_L_3_ triple helicate, thus demonstrating an anion-induced transformation through aggregation. Other anions such as ReO_4_^−^ and BF_4_^−^ were also observed to lead to the same phenomenon, but with higher concentrations required.

In comparison to helical monomer 133, hexamer 134 exhibits distinct physical properties, including aggregation-induced emission enhancement and improved water stability. Furthermore, the tertiary structure induces formation of a new central cavity, defined by a terphenyl panel from each helicate, which is able to encapsulate organic guests, with enantioselective binding observed in some cases. This study constitutes the first example of biomimetic formation of tertiary structure from metal–organic architectures, with new functions arising from the tertiary structure in a similar manner to that observed for biomacromolecules.

Anion metathesis can result in conversion between multiple structures formed from the same building blocks, as demonstrated by Sun *et al.* (Fig. 13b).^122,123^ Three different 3D Pd^II^_*n*_140_2*n*_ assemblies, Pd^II^_3_L_6_135, Pd^II^_6_L_12_138 and Pd^II^_7_L_14_139, were initially prepared from ditopic benzimidazole-based ligand 140 with the NO_3_^−^, BF_4_^−^, OTf^−^, or PF_6_^−^ salt of Pd^II^. The size and shape of the product is dictated by hydrogen-bonding interactions between the inner surface of the assembly and the anions. Taking advantage of the dynamic nature of the metal–ligand bonds, two further species, Pd^II^_4_L_8_136 and Pd^II^_5_L_10_137, were isolated *via* anion-induced transformation processes. These assemblies were obtained upon addition of HSO_4_^−^ or Mo_7_O_24_^6−^, respectively, to a solution of 139.

The authors highlighted a transformation network between five structures (Fig. 13b), in which ten different cage-to-cage conversions were driven by anion exchange. Assembly 137 was determined to be the most favoured species in this complex system, with all the other species being transformed into 137 after addition of Mo_7_O_24_^6−^. In light of these multiple transformations, the authors were able to establish a binding hierarchy as follows: Mo_7_O_24_^6−^ > NO_3_^−^ > SO_4_^2−^ > BF_4_^−^ > PF_6_^−^ ≈ OTf^−^. A subsequent study revealed that squaramide, C_4_O_4_^2−^, serves as an even stronger template than Mo_7_O_24_^6−^, to drive transformation toward Pd^II^_4_L_8_ assembly 136.^123^

Anion binding can also trigger the convergence of a mixture of cages towards a unique species (Fig. 13c).^124^ With Cd^II^(OTf)_2_, the flexible subcomponent 144 forms a mixture of assemblies 141, 142 and 143 in variable proportions depending on the aniline subcomponent chosen. Further addition of BF_4_^−^ drives the mixture to exclusively face-capped Cd^II^_4_L_4_ tetrahedron 142. In this structure the methyl group of the ligand points outward, away from the cavity, creating an inner void sufficient to accommodate BF_4_^−^. Due to the flexibility of the ligand, the templating guest is necessary to obtain a single cage. Within this system, more complex architectures such as the Cd^II^_8_L_8_ tetragonal antiprismatic cage 143 are selected through using secondary interactions between the aniline subcomponents.

**Fig. 14:**
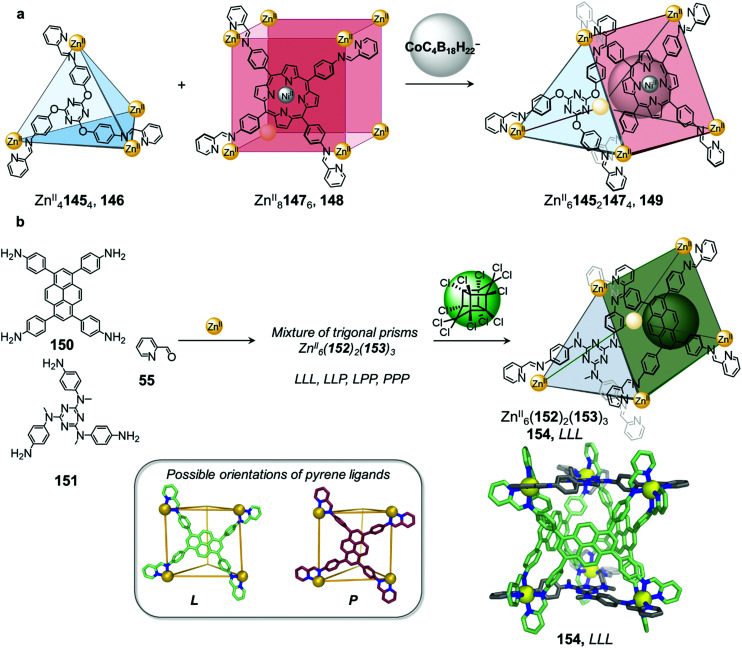
(a) Mixing tetrahedron 146 and cube 148 gives rise to the formation of triangular prism 149, templated by the anionic guest cobalticarborate (CoC_4_B_18_H_22_^−^).^92^ (b) Self-assembly of a library of up to four diastereomeric trigonal prismatic cages 154 and guest induced reconfiguration to form a single diastereomer upon addition of the pesticide Mirex. The crystal structure of cage 154 (crystallised in the absence of a guest) where all the pyrene ligands adopt the L orientation is depicted.^125^

A larger anionic guest cobalticarborate (CoC_4_B_18_H_22_^−^) templates the formation of Zn^II^_6_145_2_147_3_ triangular prism 149 from a mixture of tetrahedron 146 and cube 148 (Fig. 14a).^92^ Self-assembly of the subcomponents, to form ligands 145 and 147, required to make 146 and 148 with Zn^II^ initially leads to the exclusive formation of these homoleptic species, which only convert to heteroleptic 149 upon addition of the template. The asymmetric guest testosterone was also able to template the formation of 149. An analogue of 149 incorporating a different tritopic subcomponent forms without a template and is able to bind a wide range of natural products within its elongated cavity as well as at its periphery, illustrating the value of cage-to-cage transformations for the development of assemblies with new guest binding abilities.^92^

Very recently we demonstrated that lower symmetry rectangular building block 150 can also be incorporated with triangular building block 151 into similar Zn^II^_6_152_2_153_3_ trigonal prismatic cages (Fig. 14b).^125^ The two distinct axes of the pyrene-based ligand 153 enable it to adopt either a portrait (P) or landscape (L) orientation when capping the rectangular faces of a trigonal prismatic cage 154. The heteroleptic cage 154 forms cleanly without a template but exists as a mixture of up to four diastereomers in solution, arising from different orientational configurations of the three rectangular ligands on the cage faces. The higher symmetry diastereomers where all tetratopic ligands possess the same orientational configuration (denoted LLL and PPP) display *D*_3_ point symmetry while the isomers with a mixture of ligand orientations (denoted LLP and PPL) are of lower *C*_2_ point symmetry. The isomers also differ in cavity size and shape with the PPL isomer having a narrower and more elongated cavity relative to the LLL and LLP diastereomers, as determined by X-ray crystallographic analysis.

Although the cage panels are rigid, the different orientations that each panel can adopt enables the cage cavity to dynamically adapt to optimize the binding of guests including a family of toxic organochlorine pesticides. Incorporation of chlorinated pesticides such as Mirex results in quantitative conversion of the mixture into the LLL diastereomer, thus maximizing binding affinity to the guest. Guest molecules such as Mirex are recognized as persistent organic pollutants (POPs) and thus their selective encapsulation by 154 paves the way for the development of applications, such as sensing these toxic molecules or removing them from the environment.^22^

Metal–organic cages frequently incorporate aromatic moieties as part of their ligands. These aromatic panels not only enable the ligands to maintain the rigidity required to form discrete species, but may also help to enclose the cage cavity. Such hydrophobic cavities are segregated from the bulk solution, and thus can allow neutral guest encapsulation.

**Fig. 15:**
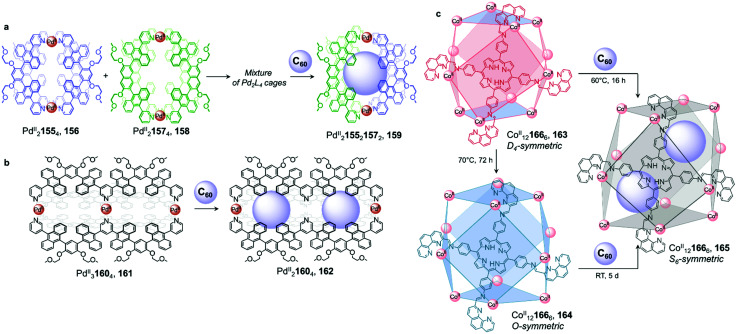
Examples of fullerene induced cage-to-cage transformations. (a) Mixing cages 156 and 158 results in the formation of a library of Pd^II^_2_L_4_ assemblies, which is driven towards unique host–guest architecture 159 by the introduction of C_60_.^126^ (b) Double cage 161 loses a Pd^II^ centre upon encapsulation of two fullerenes in order to optimise binding in 162.^128^ (c) Both cages 163 and 164 were transformed into 165 upon C_60_ encapsulation, to maximise interactions between the guest molecules and the walls of the cage.^129^

As in previous examples, neutral guest binding can also induce the self-sorting of a cage mixture, leading to the formation of a unique host–guest complex (Fig. 15a).^126,127^ Upon mixing homoleptic Pd^II^_2_155_4_ cage 156 and Pd^II^_2_157_4_ cage 158 in DMSO, Yoshizawa and co-workers observed the formation of a mixture of homoleptic and heteroleptic cages (Fig. 15a). Addition of C_60_ afforded new heteroleptic Pd^II^_2_155_2_157_2_ capsule 159 quantitatively. Calculations indicated that the *cis* isomer was lower in energy than its *trans* analogue, and therefore most likely formed preferentially. The authors inferred that aromatic stacking interactions between the large anthracene panels of the host and C_60_ were responsible for stabilisation of this complex. Similarly, using an isomerisable and desymmetrised ligand, the same group reported a study in which C_60_ drives the transformation of a larger mixture of up to 42 different isomeric assemblies toward a single host–guest complex.^126^

Further investigation by the Yoshizawa group revealed that C_60_ also induced partial demetallation of coordination cage 161 (Fig. 15b).^128^ Extending the backbone of their previous ligand to form tritopic, W-shaped 160, Pd^II^_3_160_4_ double cage 161 was isolated. The binding ability of the new cage was initially investigated with C_60_, which had previously been encapsulated in the cavity of the Pd^II^_2_L_4_ single-cage analogue 156. After heating C_60_ and 161 at 110 °C in DMSO overnight, they observed a large upfield shift of the ^1^H NMR signals corresponding to the central pyridine moieties, consistent with cleavage of the four central Pd^II^–N bonds and a loss of this metal ion to form a new Pd^II^_2_160_4_ cage 162. ESI-TOF MS analysis confirmed the formation of a host–guest complex where two fullerenes were encapsulated within 162. The sixteen aromatic panels of the final peanut-shaped cage were inferred to interact strongly enough with the two guests to eject the central Pd^II^ ion, with multiple aromatic-stacking interactions playing a crucial role in the stabilization of the coordinatively unsaturated architecture.

We described a different reconfiguration arising from fullerene encapsulation (Fig. 15c).^129^ A Co^II^_12_166_6_ cuboctahedral framework presented a high level of conformational flexibility, with structurally distinct isomers obtained under different conditions. Co^II^_12_166_6_ isomers 163 and 164 are formed upon reaction of the precursor subcomponents and Co^II^ in acetonitrile either at room temperature or by heating at 60 °C overnight, respectively. X-Ray structures indicate *D*_4_ symmetry for 163, whereas 164 is *O*-symmetric. In 164, all metal centres have the same *Δ* or *Λ* handedness, resulting in six square faces capped by the tetrakis-tridentate porphyrin ligand 166. In contrast, 163 has both *Δ* and *Λ* vertexes in a 1 : 2 ratio resulting in four rectangular faces and two square ones. Isomer 163 transforms into 164 upon heating to 70 °C.

Both cages transform into another isomer 165, with *S*_6_ symmetry, after the cooperative binding of two C_60_ fullerenes. In 165, the ligand environment is completely desymmetrised, leading to a distorted structure with equivalent proportions of *Δ* and *Λ* metal centres. The architecture adopts an axially elongated configuration to optimize both guest-guest and host–guest contacts. In this system of cage diastereomers, the rotational flexibility of the ligand phenanthroline moieties allows multiple configurations to be adopted, which is key to the plasticity of the system. The bis-fullerene adduct 165 exhibits different cooperativity and binding affinities towards peripheral anionic guests than does 164, highlighting the ability of the cage-to-cage transformation to tune the properties of an assembly without altering the connectivity of its framework.

**Fig. 16:**
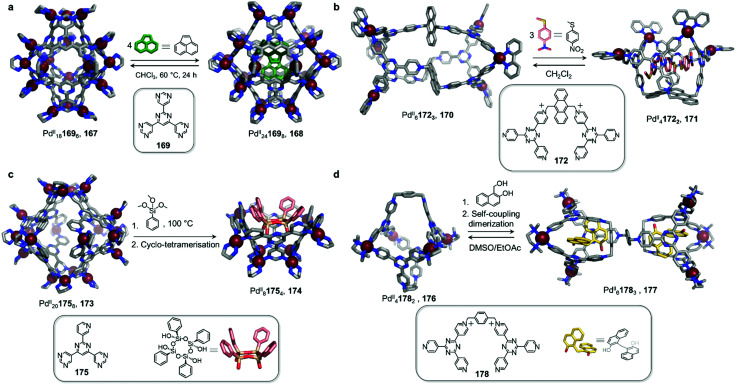
Examples of cage-to-cage tranformations induced by neutral guests in aqueous solution. (a) Binding of neutral guests induced an expansion of capsule 167 in order to maximise host–guest interactions, thus transforming 167 into 168.^128^ (b) In similar fashion, the binding of three molecules of methyl(4-nitrophenyl)sulfane triggered the transformation of cage 170 into bowl-shaped 171. This process reverses when the guest molecules were extracted from the cavity.^130^ (c) The tetramerization of the trialkoxysilane guest within the cavity of 173 induced the transformation of this host into new species 174.^131^ (d) Cage 176 transformed into double cage 177 when the guest underwent a self-coupling dimerization after encapsulation.^132^

Work from the Fujita group also highlights the ability of neutral guests to induce cage-to-cage conversions (Fig. 16a).^128^ Whereas previously-discussed examples have consisted of transformations taking place in organic solvents, this study was conducted in water. The pyrimidine-based ligand 169 assembles with *cis*-capped Pd^II^ to produce Pd^II^_18_169_6_ trigonal bipyramidal cage 167. This well-enclosed structure presents a hydrophobic inner cavity suitable for large neutral guests.

An unexpected transformation of the architecture occurs upon addition of excess acenaphthylene (Fig. 16a). Single-crystal analysis revealed the formation of a new and larger Pd^II^_24_169_8_ octahedral host 168, possessing an expanded cavity (943 Å^3^, compared to 381 Å^3^ for 167) where four guest molecules were accommodated with strong aromatic-stacking interactions between the electron-rich guest and the electron-deficient ligand panels. Calix[4]arene, as well as its linear tetra-phenol analogue, also induce transformation of 167 to 168, whereas a smaller tri-phenol does not induce transformation. Removal of the guests is only possible by heating the host–guest complexes of 168 in chloroform at 60 °C for 24 h, resulting in regeneration of 167.

In a similar fashion, the Sun group demonstrated a reversible guest-induced cage-to-cage transformation. They first synthesized tetratopic dicationic ligand 172, with a bulky central anthracene linker (Fig. 16b).^130^ After addition of *cis*-protected Pd^II^ and self-assembly in water, the *D*_3_-symmetric Pd^II^_6_172_3_ capsule 170 assembles. This new architecture is able to bind a series of adamantane guests in a 1 : 8 host–guest ratio.

Surprisingly, when methyl(4-nitrophenyl)sulfane was added to a solution of host 170, the authors observed a modification of the ^1^H-NMR spectra, suggesting a transformation of the structure upon guest encapsulation. The X-ray structure of the host–guest complex revealed a guest-adaptive transformation, where cage 170 was converted into a *C*_2v_-symmetric Pd^II^_4_172_2_ bowl-shaped assembly 171, in which three guests were accommodated. Aromatic stacking interactions were observed between the three planar guests and the electron deficient ligand panels. The transformation reverses following removal of the guest through extraction with CH_2_Cl_2_, or by addition of excess 1-adamantanecarboxylic acid.

Fujita and co-workers designed asymmetric ligand 175, which forms Pd^II^_20_175_8_ capsule 173 (Fig. 16c). This architecture is more flexible than the previously-described Pd^II^_24_L_8_ capsule 168, and can encapsulate a large variety of guests.

However, upon encapsulation of a large guest, the authors observed a remarkable capsule-to-bowl conversion. When phenyl trimethoxysilane condenses into the cyclic tetrasiloxane derivative shown in Fig. 16c, the cage splits into two Pd^II^_8_175_4_ pyramid-shaped bowls 174. This transformation releases four Pd^II^ centres and leads to a maximisation of host–guest interactions at the expense of metal–ligand bonds. Remarkably, because of the template effect of 174, a single all-*cis* stereoisomer of the cyclic tetrasiloxane is formed stereoselectively.^131^

Sun and co-workers demonstrated a similar phenomenon, where the reaction of entrapped guests was responsible for capsule transformation (Fig. 16d).^131,132^ Pd^II^_4_178_2_ cage 176, featuring a large internal cavity, undergoes transformation to a new Pd^II^_6_178_3_ cage 177, which features two independent cavities, following self-coupling of the guest. First, water-soluble cage 176 was found to bind four 1-hydroxymethyl-2-naphthol molecules inside its central pocket. However, after heating this mixture, modifications to the ^1^H NMR spectrum of the complex were observed. X-ray analysis determined the structure of unprecedented Pd^II^_6_178_3_ product 177. Modification of the guest was also revealed by this experiment, indicating the formation of two 2,2′-dihydroxy-1,1′-dinaphthylmethane guests from dimerisation of the initially-added guest. The final host presented a surprising structure, in which two 178 ligands have the same *cis* configuration as in 176, while the third one is found to adopt a *trans* configuration to bridge two separated cavities where the guests were encapsulated.

The strong aromatic stacking interactions observed between the naphthalene rings of the two guest molecules and the 2,4,6-tris(4-pyridyl)-1,3,5-triazine panels of the cage were inferred to provide the principal driving force for the induced-fit cage transformation. In contrast to the examples of cage fusion discussed in Section 2.4, this process could be considered a cavity fission or ‘mitosis’, as described by the authors. The cage transformation process reverses after dissolving the host–guest complex of 177 in DMSO, leading to guest release and regeneration of the initial Pd^II^_4_178_2_ cage 176, which can be recycled through precipitation by EtOAc.^132^

The four examples shown in Fig. 16 are archetypal examples of coordination cages displaying induced-fit behaviour, reminiscent of that of enzymes, which can change their conformations and shapes to fit a target substrate. In each case the binding of small molecules leads to a recombination of the cage components, allowing the incorporation of several substrate molecules within the cavity. Moreover, the reactions of guests within 174 and 177 represent an important step towards mimicking the induced-fit catalysis of enzymes in artificial systems. The reversibility of these transformations upon guest extraction, also enabled by the dynamic nature of their Pd^II^-pyridine bonds, paves the way towards achieving catalytic processes inside the inner voids of adaptable metal–organic hosts.

**Fig. 17:**
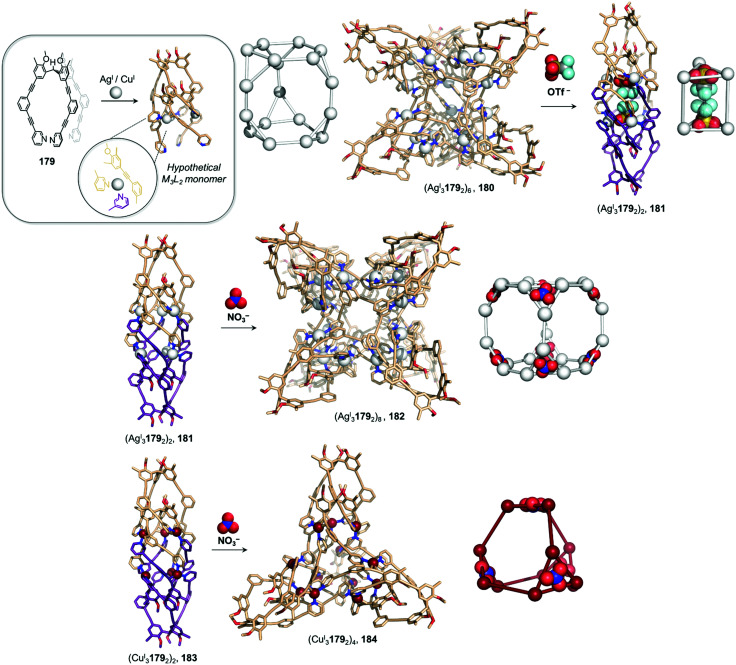
Anion-driven reconfiguration of a new class of metal–organic assemblies: polyhedral links. Component flexibility, as well as secondary π-coordination between the alkyne linkers of the ligand and the metals, allow the formation of highly entangled architectures. Strong templation effects were shown to drive the transformation between the different architectures.^133,134^

Recent work by Fujita and co-workers has shed light on a remarkable new class of intricate, highly entangled metal–organic assembly capable of reconfiguration upon simple anion exchange (Fig. 17).^133,134^ In 2019, they first reported the use of tripodal ligand 179 which coordinates to metal ions *via* two distinct coordination modes. The pyridyl donors coordinate alongside the alkyne moieties of the ligand, resulting in simultaneous σ- and π-coordination to either Cu^I^ or Ag^I^. This bonding arrangement favours the formation of a capped double-propeller M_3_179_2_ (M = Cu^I^ or Ag^I^) subunit, in which the two organic ligands are entangled and the three metal ions are each coordinated to a pyridyl donor of the outer ligand and an acetylene donor of the inner ligand. These metal–organic building-blocks can thus come together to form larger assemblies with a (M_3_179_2_)_*n*_ structure, where the vacant coordination site of each metal ion is coordinated to a free pyridyl donor from an outer ligand of another M_3_L_2_ moiety. Different topologically complex architectures were obtained by varying the self-assembly conditions, including (M_3_179_2_)_2_ interlocked cages 181 and 183, (M_3_179_2_)_4_ truncated tetrahedron 184, and (M_3_179_2_)_6_ truncated trigonal prism 180, with the faces of 184 and 180 exhibiting trefoil knot and Solomon link motifs respectively.^133^

The authors explored a series of guest-triggered transformations between these oligomers driven by favourable anion templation effects.^134^ The BF_4_^−^ salt of hexameric cage 180 (with M = Ag^I^) transforms into dimer 181 upon addition of OTf^−^. The authors inferred that the bulkiness of the triflate anion prevented its incorporation into architecture 180, leading to destabilization of the assembly and inducing the formation of 181. The same dimeric capsule 181 is also formed in the presence of BF_4_^−^. Exchange of BF_4_^−^ by NO_3_^−^ produces larger (Ag^I^_3_179_2_)_8_ octameric truncated cube 181. Single-crystal X-ray analysis revealed the structure of the Ag^I^_24_179_16_ assembly, with overall *O*-symmetry. NO_3_^−^ incorporation leads to a contraction of the Ag^I^_3_L_2_ subunits through binding to the Ag^I^ centres, enhancing the stability of the overall assembly, which is entropically disfavoured as compared to the smaller oligomers. It is worth noting that assembly 182 could not be obtained by direct self-assembly from its components. The authors inferred that transformation takes place without full dissociation of the Ag^I^_3_179_2_ subunits, preventing the precipitation of Ag^I^NO_3_, which occurs upon direct mixing of Ag^I^NO_3_ and 179. Interestingly, an analogous nitrate-induced transformation of dimer 183 yields truncated tetrahedron 184 instead of the octameric species when Cu^I^ is used in place of Ag^I^, indicating NO_3_^−^ does not have the same templating effect in this case.

### 3.2. Concentration-induced transformations

The addition of external species, such as new components or guests, is not always necessary for cage-to-cage transformations to occur. The combination of flexible ligands with labile metal ions can allow a diverse range of architectures to form under different self-assembly conditions. In such cases the product observed under a given set of conditions is governed by the interplay of entropy and enthalpy for systems under thermodynamic control. Structures can thus be interconverted by concentration changes according to Le Chatelier's principle, with higher nuclearity structures usually favoured at higher concentrations. Newkome^135^ has drawn an analogy between such concentration-dependent cage transformations and the fission-fusion process in biological systems.^120^

Newkome and co-workers have greatly contributed to the development of concentration driven cage-to-cage transformations, exploiting the coordination of terpyridine-based organic building blocks with octahedral metal ions. Early studies reported the concentration-dependant switching from a planar *bis*-rhombus assembly to a tetrahedron,^135,136^ and the cage-to-cage conversion from a cuboctahedron to an octahedron.^136^

**Fig. 18:**
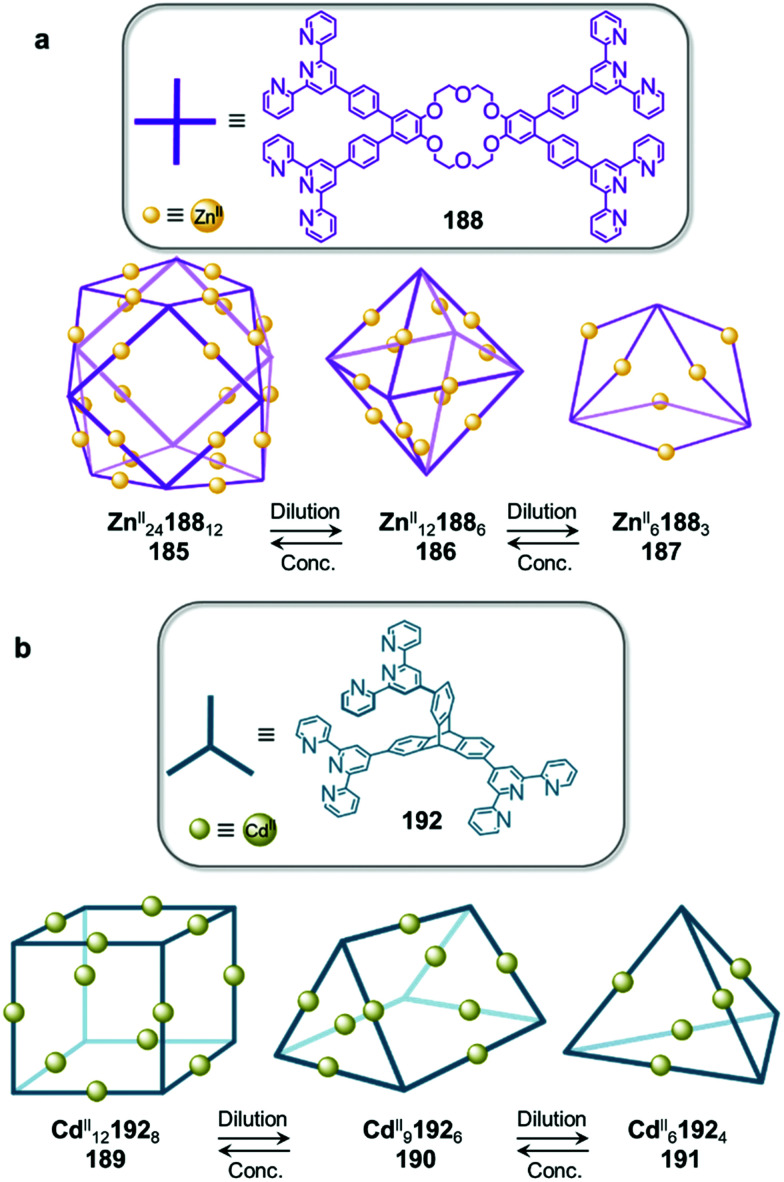
Concentration driven transformations. (a) Three architectures based on crown-ether ligand 188 and Zn^II^ interconvert depending on concentration.^137^ (b) In a similar manner, the reaction of triptycene ligand 192 and Cd^II^ produces three transformable species.^136^

Building on the success of these two studies, they developed systems consisting of three interconverting cages (Fig. 18).^137^ In 2016, they highlighted the ability of terpyridine-decorated crown ether ligand 188 to switch between three distinct assemblies, Zn^II^_24_188_12_ cuboctahedron 185, Zn^II^_12_188_6_ octahedron 186, and Zn^II^_6_188_3_ bis-triangular complex 187, upon variation of the concentration (Fig. 18a). The flexibility permitted by the 18-crown-6 moiety is critical to the preparation of the different structures. Indeed, the dihedral angle between the two benzene rings can vary between 0° and 127°, thus allowing cages 185, 186, and 187 to be formed. A second study described another concentration-dependant system of three architectures, this time using a more rigid triptycene-centred ligand 192. Upon reaction of this ligand with labile Cd^II^, Cd^II^_12_192_8_ cube 189, Cd^II^_9_192_6_ prism 190, and Cd^II^_6_192_4_ tetrahedron 191 were isolated and interconverted as a function of concentration (Fig. 18b).^136^ In both studies, dilution led to cage fission into smaller and more entropically-favourable architectures. Conversely, an increase in concentration drove the system towards the formation of larger structures *via* cage fusion processes.

**Fig. 19:**
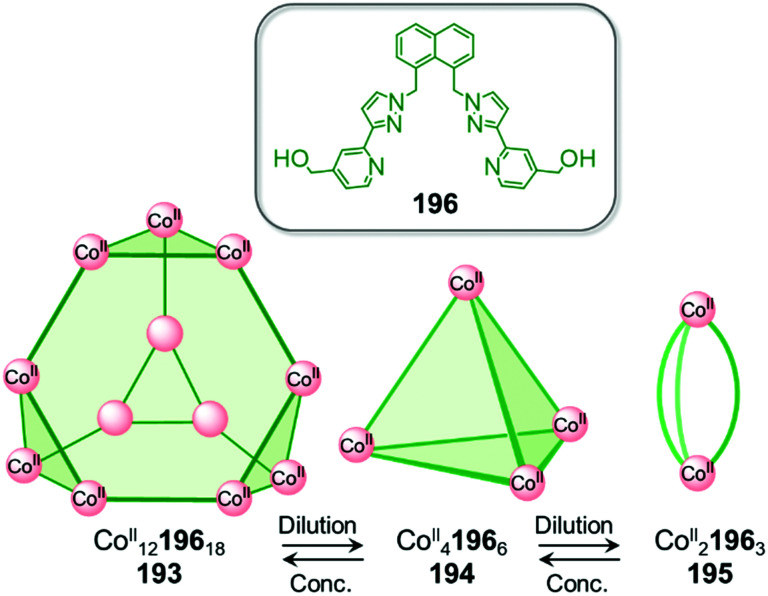
Ward and co-workers also demonstrated that concentration and hydrophobic effects drive cage transformations among a series of Co^II^_2*n*_196_3*n*_ architectures.^138^

Exploiting entropic factors, Würthner^70^ and Ward^138^ have also developed concentration-dependant transformations from one complex to another. Würthner's group reported a perylene bisimide-edged Zn^II^_4_L_6_ tetrahedron that converted to a smaller Zn^II^_2_L_3_ helicate on dilution. Ward *et al.* described a more elaborate system, consisting of three different assemblies, Co^II^_12_196_18_ truncated tetrahedron 193, Co^II^_4_196_6_ tetrahedron 194 and Co^II^_2_196_3_ mesocate 195, (Fig. 19), which interconvert in aqueous solution following concentration and temperature changes.^138^ Decreasing the concentration or increasing the temperature gives a higher proportion of the entropically-favoured smaller assemblies, while high concentrations and low temperatures favour the largest assembly.

The authors also postulated that the hydrophobic effect plays a crucial role in the formation of the larger architectures. Reorganization of the smaller cages into larger complexes decreases the surface area to volume ratio, allowing more of the surfaces of the hydrophobic ligands to be shielded from the aqueous environment. This hypothesis is supported by the observation that the smallest assembly 195 is obtained as the only detectable product in non-aqueous nitromethane solvent.

**Fig. 20:**
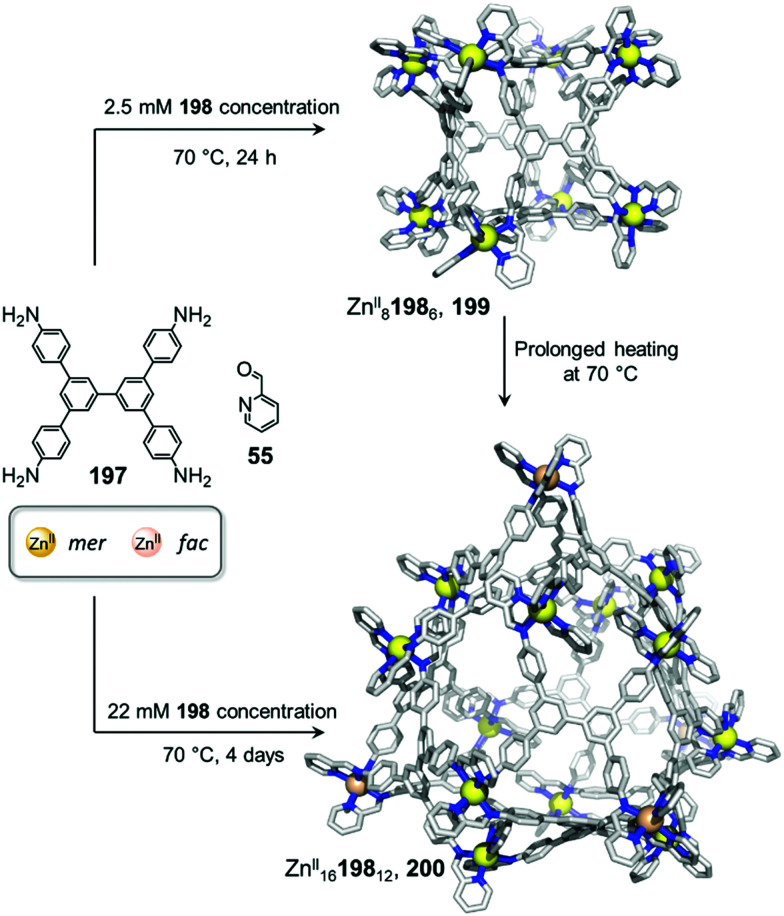
Concentration dependent formation of a Zn^II^_8_198_6_ cube-like assembly 199 and unprecedented Zn^II^_16_198_12_ structure 200 with *fac* Zn^II^ centres are shown in orange and *mer* Zn^II^ in yellow. Structure 199 converts into 200 after heating.^139^

Concentration can also play a role in systems that are not in thermodynamic equilibrium, as demonstrated recently by our group. Self-assembly of a twisted rectangular subcomponent 197 with 2-formylpyridine 55, forming ligand 198, and Zn^II^ yielded an unprecedented Zn^II^_16_198_12_ structure 200 at an initial ligand concentration of 22 mM (Fig. 20).^139^ The structure consists of four ‘half-cube’ units joined together by *mer* Zn^II^ centres, with each unit crowned with a *fac* Zn^II^ centre that corresponds to the vertex of an extended tetrahedron with overall *T* point symmetry. The structure of 200 is reminiscent of that of the protein capsid formed by *Archaeoglobus fulgidus* ferritin, with the ligands of 200 mapping onto dimeric protein subunits of the ferritin.

When the initial ligand concentration is reduced to 2.5 mM, a simpler Zn^II^_8_L_6_ cube-like architecture 199, with eight *fac* Zn^II^ centres, is obtained. The smaller capsule 199 converts to the larger capsule 200 following heating to 70 °C. This structural conversion even takes place at low concentrations, albeit slowly, allowing us to infer that 200 is the thermodynamically favoured product, with 199 being an isolable kinetic product. Unlike the previously-discussed examples of systems operating under equilibrium, the conversion of 199 to 200 is irreversible.

Unlike that of many large cages, the cavity of 200 is sufficiently enclosed to bind guests and the structure was observed to bind multiple equivalents of the Mo_6_O_19_^2−^ anion, rendering it one of the largest reported cages capable of guest binding. In contrast, no interaction was observed between the smaller cube-like cage 199 and the same anion. However, the presence of Mo_6_O_19_^2−^ accelerated the conversion of 199 into 200.

### 3.3. Solvent- and pH-induced transformations

Solvent choice can drive structural reorganisation of coordination cages, acting as an external stimulus. In most cases, reconfiguration of the architecture is due to solvent-dependent supramolecular interactions such as hydrogen bonds or the hydrophobic effect in aqueous media. Pioneering work from Fujita,^138^ Lehn^140,141^ and Williams^142^ on solvent-dependant metallo-supramolecular reassembly established how changes in solvent polarity could lead to transformation between structures. In other instances, solvent molecules can act as guests within structures.

**Fig. 21:**
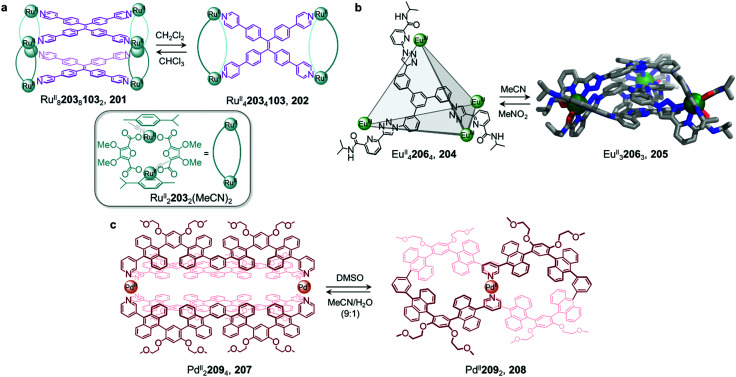
Examples of solvent-induced transformations. (a) Reversible cage-to-macrocycle transformation induced by a switch between CHCl_3_ and CH_2_Cl_2_.^143^ (b) Cage 204 and sandwich-like architecture 205 interconvert by switching the solvent from MeCN to NO_2_Me.^144^ (c) Interconversion between ‘peanut’ cage 207 and butterfly complex 208, driven by changing between DMSO and a mixture of MeCN/H_2_O.^145^

In 2012, Severin *et al.* reported a striking example of this phenomenon, where dramatic structure modifications arose from subtle solvent modifications (Fig. 21a).^143^ The assembly of Ru^II^ metallacrown complex Ru^II^_2_203_2_(MeCN)_2_ and tetra(pyridyl) TPE ligand 103 in chloroform first results in the formation of Ru^II^_8_203_8_103_2_ rectangular prism 201*via* replacement of the weakly coordinating acetonitrile molecules with the stronger pyridyl donors of 103. Notably, switching the solvent from chloroform to dichloromethane leads to the formation of planar rectangular Ru^II^_4_203_4_103 structure 202.

The crystal structure of 202 provides an explanation for this phenomenon by showing that two CH_2_Cl_2_ molecules bind in the cavity of each metallacrown moiety, interacting with the oxygen atoms linked to the Ru^II^ centres *via* C–H⋯O hydrogen bonds, thus resulting in an enthalpic stabilization of the structure. The solvent-induced interconversion between 201 and 202 is fully reversible, suggesting that each complex is the thermodynamic product in each respective solvent. The interaction of 201 with CH_2_Cl_2_ disturbs the finely balanced energetics of the system, where 202 is entropically favoured but exhibits enthalpically-unfavourable ligand strain.

More recently, Sun and co-workers have observed a solvent-controlled interconversion between lanthanide-based metal–organic assemblies (Fig. 21b).^144^ Eu^III^_4_206_4_ tetrahedron 204 is obtained in nitromethane, whereas sandwich-like Eu^III^_3_206_3_ structure 205 is isolated in acetonitrile. Changing the solvent successfully drives transformation of one cage into the other. The authors inferred that subtle differences in solvent polarity were responsible for this observation.

Pd^II^-based coordination cages reported by the Yoshizawa group also underwent solvent-driven interconversions (Fig. 21c).^145^ Peanut-like metal–organic cage 207 can be obtained from W-shaped dipyridyl ligand 209 in a 9 : 1 mixture of acetonitrile and water. Initially-formed 207 has a structure related to its pyridine analogue 160, previously isolated after fullerene encapsulation, with 207 also binding two C_60_ guests. Switching the MeCN/H_2_O solvent mixture to DMSO results in the transformation of 207 into Pd^II^209_2_ complex 208, consisting of two slightly twisted tubes linked together around a central Pd^II^ ion. This novel assembly no longer binds fullerenes due to its smaller and less well-defined cavities. The more enclosed assembly 207 was inferred to be favoured in aqueous organic solution due to the hydrophobic effect, whereas entropically favoured 208 was formed in DMSO.

**Fig. 22:**
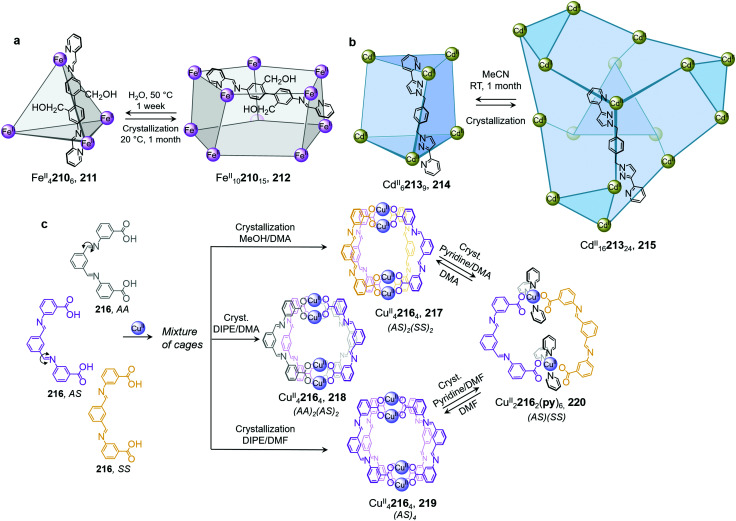
Examples of solvent-influenced transformations during crystallisation. (a) Kinetically trapped prism 212 is obtained through crystallisation of a solution of 211 or by modification of the reaction conditions. Dissolution of this assembly in water led to the recovery of tetrahedral cage 211.^146^ (b) In a similar manner, giant assembly 215 was obtained from a solution of 214 after crystallization. However, after a long period in solution, crystals of 215 were observed to transform back into trigonal prism 214. (c) Crystallization allowed the selection of specific isomers from a library of cages depending on the conditions used, and introduction of pyridine induced transformation of the cages into macrocycle 220.^148^

Solubility can influence the transformation of coordination cages, especially *via* selective crystallisation (Fig. 22a).^146^ Fe^II^_4_210_6_ tetrahedron 211 and a Fe^II^_10_210_15_ pentagonal anti-prism 212 are prepared from the same subcomponents, but interconvert depending on the conditions. Tetrahedron 211 was first synthesized by mixing its building blocks in water at 50 °C. Surprisingly, attempts to grow crystals of this species from aqueous media resulted in the isolation of 212 only. This prismatic cage was also found to be water soluble, but was less so than the tetrahedron, thus explaining its preferential crystallization.

Equilibration between the two species in solution enables complete conversion of 211 to 212*via* crystallisation. A 9 : 1 mixture of methanol/water at room temperature provides optimal conditions for forming the larger architecture in solution. While the conversion of 212 back to 211 is not observed in solution at room temperature, prolonged heating at 50 °C for one week results in regeneration of the smaller assembly. We inferred that 212 was in fact a kinetic product, trapped due to the large number of metal–ligand bonds holding it together, with 211 being the thermodynamically-favoured species.

In an earlier study, Ward *et al.* had observed a similar phenomenon upon crystallization and dissolution (Fig. 22b) of Cd^II^_16_213_24_ tetra-capped truncated tetrahedron 215.^147^ Dissolution of crystalline 215 led to significant differences in the NMR spectra after a few weeks at room temperature or two days at 60 °C. Careful examination of the ^1^H NMR spectrum, as well as DOSY experiments, led the authors to unambiguously confirm the formation of Cd^II^_6_213_9_ trigonal prismatic cage 214 as the major product. The authors noted that both structures are based on different combinations of triangular Cd^II^_3_L_3_ panels, such that rearrangement of the larger cage into the smaller one may proceed *via* partial dissociation into and recombination of intermediates in which the Cd^II^_3_213_3_ panels are conserved. The two species were in slow equilibrium with 214 predominant in solution, while crystallisation promoted formation of 215. In both this example and the previous one, entropic factors drove transformations toward the favoured species in solution.

Bloch and co-workers have recently demonstrated self-sorting of a dynamic combinatorial library upon crystallization (Fig. 22c), driven by solubility and subtle crystal packing effects.^148^ They used subcomponent self-assembly to create a dicarboxylate ligand 216 through double imine condensation between isophthalaldehyde and 3-aminobenzoic acid, which then assembled with Cu^II^ to form Cu^II^_4_216_4_ cages based on dicopper paddle-wheel nodes. The ligand adopted three different rotational conformers (216^AA^, 216^AS^, 216^SS^) depending on the *anti*- (A) or *syn*- (S) configuration of the arms with respect to the benzene core. Although 34 capsule isomers are possible, three distinct assemblies were selectively crystallized using different crystallisation solvents.

Vapour diffusion of methanol into dimethylacetamide (DMA) afforded *trans*-[Cu^II^_4_216^SA^_2_216^AA^_2_(DMA)_4_] capsule 217, while the use of diisopropyl ether (DIPE) as a co-solvent led to the formation of Cu^II^_4_216^SA^_4_ capsule 218. Similarly, vapour diffusion of DIPE into a DMF solution yielded *trans*-[Cu^II^_4_216^SA^_2_216^SS^_2_(DMF)_4_] capsule 219. DFT calculations suggested that none of these structures was the lowest energy isomer, suggesting that self-sorting and crystallization occurred simultaneously.

Another solvent-driven reconstitution is observed when 217 or 219 is dissolved in a 3 : 7 pyridine/DMF mixture, leading to the formation of a new [Cu^II^_2_216^SS^216^AS^(pyridine)_6_] macrocycle 220. Contrary to the Cu^II^_2_ paddle-wheels of the capsules, in the macrocycle the carboxylate ligands adopt a monodentate binding mode, bridging the two Cu^II^ centres, the square-pyramidal coordination spheres of which were each completed by three pyridine ligands. These transformations were found to be reversible, with recovery of the cage structures upon dissolution and heating of the macrocycle in DMA or DMF.

A similar phenomenon was observed by Clever and co-workers, with the selective crystallization of three different species, a Pd^II^_3_97_6_ ring, a Pd^II^_4_97_8_ tetrahedron, and a Pd^II^_6_97_12_ octahedron obtained from a single fluorenone-containing ligand 97. Although these three architectures were in equilibrium in acetonitrile, changing the conditions and the solvents for crystallization provided access to each unique species, allowing X-ray structural analysis to unambiguously confirm the existence of the three assemblies.

In addition to solvent, pH and ligand basicity also constitute external stimuli that can give rise to structural modifications of metal–organic structures. For example, studies by Hardie,^149^ Chand,^148^ and Crowley^150,151^ have reported the 4-dimethylaminopyridine (DMAP) induced disassembly of Pd^II^-based metal–organic cages, highlighting the high stability of [Pd^II^(DMAP)_4_]^2+^, a consequence of the great donor strength of DMAP. We also studied the influence of pH on the assembly and disassembly of tetrahedral cages for cargo uptake and release.^152^ However, the systematic influence of ligand basicity on the stability of supramolecular assemblies has not been widely studied.

**Fig. 23:**
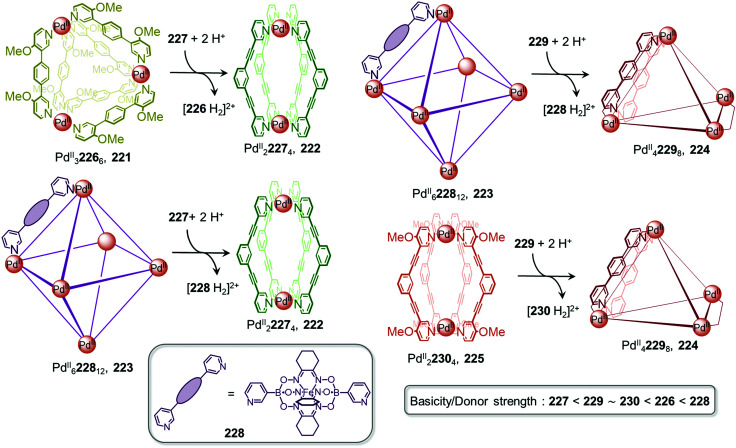
Acid-driven transformation between assemblies based on basicity and donor strength of the ligands of the system.^153^

To gain new insights into this phenomenon, Severin and co-workers studied the impact of subtle basicity differences between five pyridine-based ligands 226–230 (Fig. 23).^153^ The relative basicities and donor strengths of the five ligands were initially determined by NMR titration with trifluoroacetic acid (TFA) and evaluation of the Huynh Electronic Parameter,^154^ respectively. These parameters were correlated, with both increasing in the order 227 < 229–230 < 226 < 228. These organic building-blocks were found to form distinct Pd^II^_*n*_L_2*n*_ structures, 221–225. Competition experiments involving the addition of pyridine or TFA revealed an inverse relationship between the stability of the cage in the presence of acid and pyridine. Cages prepared from ligands with low basicity/donor strength were most susceptible to pyridine-induced disassembly, but most stable to acid. This contrasting stability enables five different acid-induced cage-to-cage transformations to be realised in the system. The more acid-sensitive octahedron 223 transforms into capsule 222 or tetrahedron 206. Similarly, ring 203 converts into the more stable 224, while capsule 225 becomes 224.

## 4. Multi-stimuli responsive transformation networks

In the previous sections we have highlighted cage-to-cage transformations induced by a single type of stimulus. Reports of transformation between distinct metal–organic structures in response to multiple stimuli are rarer. The application of multiple stimuli can allow access to new products that cannot be obtained using individual stimuli alone, or allow pathway-dependent behaviour to emerge in multi-stimuli responsive networks. This increase in complexity allows synthetic supramolecular systems to approach the functionality of their biological counterparts, which are extremely sensitive to a broad range of stimuli. The multi-stimuli responsive networks reported to date fall under two main categories: unique cage-to-cage transformations induced by multiple stimuli, or multiple stimuli giving rise to different transformation products.

**Fig. 24:**
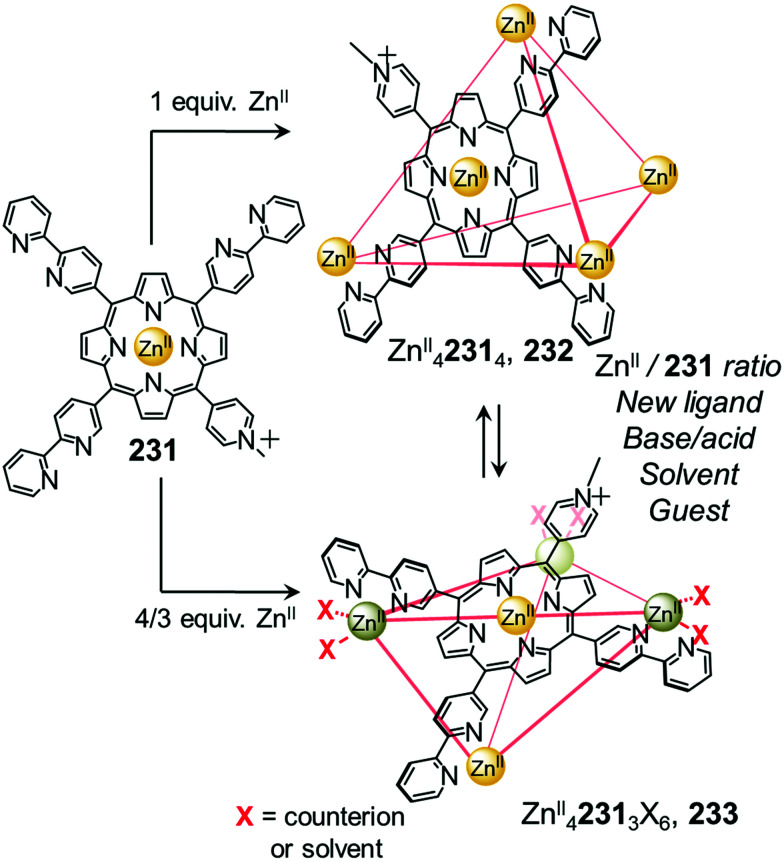
Interconversion between tetrahedron 232 and bowl-shaped 233 triggered by five distinct stimuli.^155^

Recently, the Shionoya group reported a single transformation that could be triggered by five distinct stimuli (Fig. 24).^155^ Two different structures with different stoichiometries, Zn^II^_4_231_4_ tetrahedron 232 and bowl-shaped Zn^II^_4_231_3_X_6_ (X = solvent or anion) 233 form in equilibrium from Zn^II^ and a simple zinc-porphyrin based ligand 231 with three bidentate binding sites. In addition to altering the metal–ligand stoichiometry, the two cages interconvert following the introduction of a third ligand, modification of the pH or solvent, or through the addition of a guest. The difference in stoichiometry between tetrahedron 232 and bowl-shaped 233 is crucial to their interconversion. Addition of phenanthroline to bowl-shaped 233 results in transformation to tetrahedron 232 as a result of sequestration of Zn^II^ ions as thermodynamically stable [Zn^II^(phen)_3_]^2+^. Addition of Br^−^ as a ligand also favours 232; conversely, 233 is produced from 232 incorporating bromide after treatment with Ag^I^OTf. Likewise, addition of *N,N*-diisopropylethylamine to 233 leads to the removal of Zn^II^ from the equilibrium as Zn^II^(OH)_2_, and formation of 232, a process which reverses through addition of TFA. Aqueous solvent also favours 232.

The two assemblies also interconvert *via* addition of adamantane or a sulfonamide derivative, which are good guests for 232 or 233, respectively. Finally, addition of an outward-facing ligand to 233 induces the uptake of a weakly binding guest by 233, which was unable to drive the transformation on its own. This ability to use multiple stimuli to trigger a single transformation could find use in multi-responsive materials and more complex networks, where orthogonal stimuli are needed to prevent an effect on other network components.

**Fig. 25:**
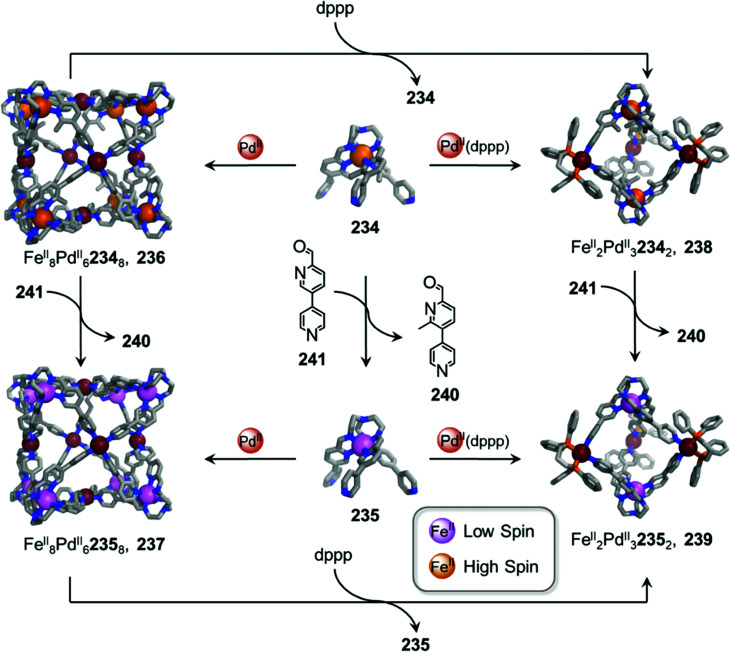
Stepwise self-assembly and structural transformations between heterobimetallic cages 236–239.^156,157^

Distinct stimuli more often give different transformation products, which can allow orthogonal transformations between structures to be achieved. Lützen and co-workers have illustrated this concept using a network controlled through the introduction of competing metal centres and subcomponents (Fig. 25), which influence either the cage structure or its spin state.^156^ Mononuclear metallo-ligands 234 and 235^157^ were initially prepared through subcomponent self-assembly of tren and Fe^II^ with 240 and 241, respectively (Fig. 25). Further self-assembly of both metallo-ligands with 1.5 equiv. of [Pd^II^(MeCN)_4_](BF_4_)_2_ leads to the formation of cubic cages 236 and 237, while reaction with 0.75 equiv. *cis*-protected [(dppp)Pd^II^(OTf)_2_] (dppp = 1,3-bis(diphenylphosphino)propane) leads to the assembly of trigonal-bipyramidal cages 238 and 239.

The Fe^II^ centres of all complexes based on 240 display a high-spin (HS) configuration as a consequence of steric hindrance arising from the methyl substituents, while those based on 241 exhibit a low-spin (LS) configuration. In all cases the HS assemblies convert to their LS analogues following subcomponent exchange. Addition of the less hindered ligand 241 brings about transformation driven by alleviation of steric strain. The cubic cages 236 and 237 convert into bipyramidal cages 238 and 239, accompanied by the release of excess metallo-ligand, *via* addition of the chelating phosphine dppp, this time with conservation of spin state. The distinct chemical stimuli used in this system thus allow either the magnetic or structural properties of the assemblies to be altered in a controlled manner.

**Fig. 26:**
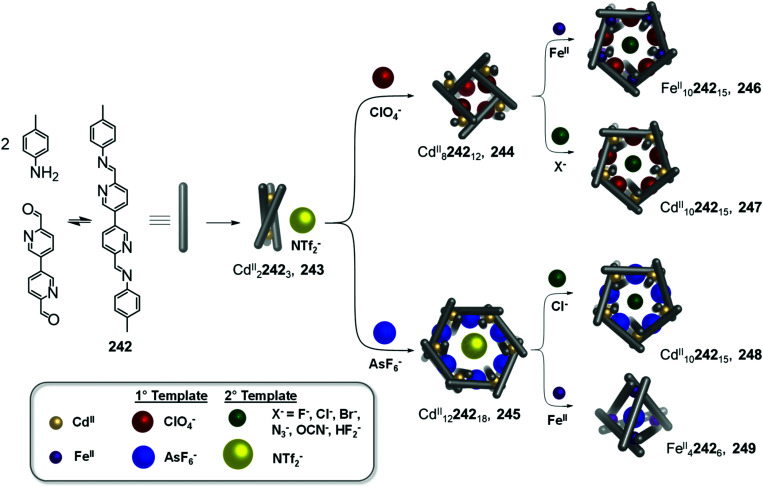
Anion- and metal-ion directed structure interconversion pathways in a network.^158^ Adapted from ref. 158 with permission from American Chemical Society, copyright 2021.

We have also developed transformation networks where chemical stimuli trigger diverse cage-to-cage conversions. The system shown in (Fig. 26) is based on five distinct architectures assembled from a single ditopic 4,4′-diformyl-3,3′-bipyridine subcomponent, which rearrange in response to both anionic and cationic signals or changes in concentration.^158^ Starting from Cd^II^_2_242_3_ helicate 243, prepared with triflimide as the only anion present, the introduction of the template ClO_4_^−^ or AsF_6_^−^ leads to transformation into a Cd^II^_8_242_12_ distorted cuboid 244 or Cd^II^_12_242_18_ hexagonal prism 245, respectively. In both cases the anionic templates bind strongly in pockets within the product framework, and were described as primary anion templates as their presence alone is sufficient to induce transformation. The conversion between helicate 243 and prism 245 is also concentration-dependent, with higher concentrations favouring the larger prism.

Both of the initially-obtained structures are further transformed upon subsequent addition of either another secondary anionic template, or displacement of Cd^II^ by more strongly-coordinating Fe^II^ centres. A series of small spherical or linear anions (X = F^−^, Cl^−^, Br^−^, N_3_^−^, OCN^−^ or HF_2_^−^) triggers the conversion of distorted cuboid 244 into X⊂Cd^II^_10_242_15_ pentagonal prisms 247, with transformation driven by the binding of these secondary templates within a central binding pocket in the pentagonal-prismatic structure. Addition of Cl^−^ as a secondary template also transforms AsF_6_^−^ templated hexagonal prism 245 into pentagonal prism 248. This structure only forms *via* sequential cage-to-cage transformation, highlighting the importance of this stepwise process for the creation of unexpected architectures.

Smaller and less labile Fe^II^ cations are able to displace the larger Cd^II^ due to the greater strength of the resulting Fe^II^–N coordination bonds with the final structure, depending on the anionic templates already present in the system. Thus, Fe^II^_10_L_15_ pentagonal prism 246 forms in the presence of ClO_4_^−^, while tetrahedral cage 249 forms in the presence of AsF_6_^−^. This system thus exhibits distinct responses to different combinations of stimuli and demonstrates the utility of metal exchange in accomplishing complex structural interconversions.

**Fig. 27:**
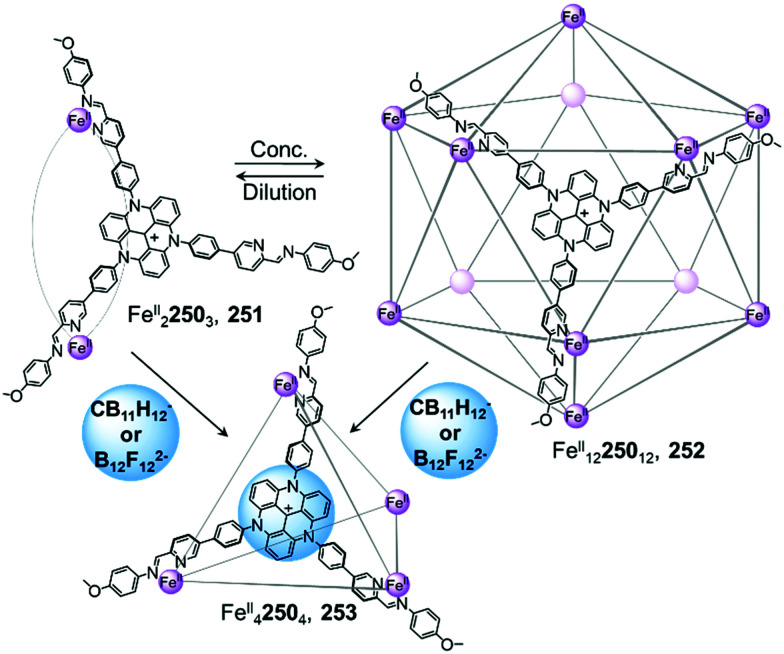
Transformations between pseudo-icosahedron 252, helicate 251, and tetrahedron 253.^159^

We recently described transformations between three different self-assembled architectures based upon a single tritopic pyridyl-aldehyde subcomponent (Fig. 27).^159^ Concentration-dependent self-assembly behaviour is also observed in this case, where a higher concentration of the triazatriangulenium-based subcomponent favours the formation of Fe^II^_12_250_12_ pseudo-icosahedron 252, while Fe^II^_2_250_3_ helicate 251 forms exclusively at a lower concentration. Conversion of either assembly into Fe^II^_4_250_4_ tetrahedron 253 occurs upon addition of a large template anion, such as CB_11_H_12_^−^ or B_12_F_12_^2−^. Large pseudo-icosahedral cage 252 may be favoured over tetrahedron 253 due to Coulombic repulsions between the cationic triazatriangulenium panels in the tetrahedron, an effect overcome by the presence of the templating anions. The same triazatriangulenium backbone was used in a previous study to construct a tetrahedral cage capable of binding nucleotide guests^160^ in water. The fluorescence of the subcomponent was conserved in the self-assembled architecture, enabling the fluorimetric recognition of guests at low concentrations. This observation suggests that water-soluble versions of the much larger cage 252 could recognise larger biomolecules, such as proteins or nucleic acids.

**Fig. 28:**
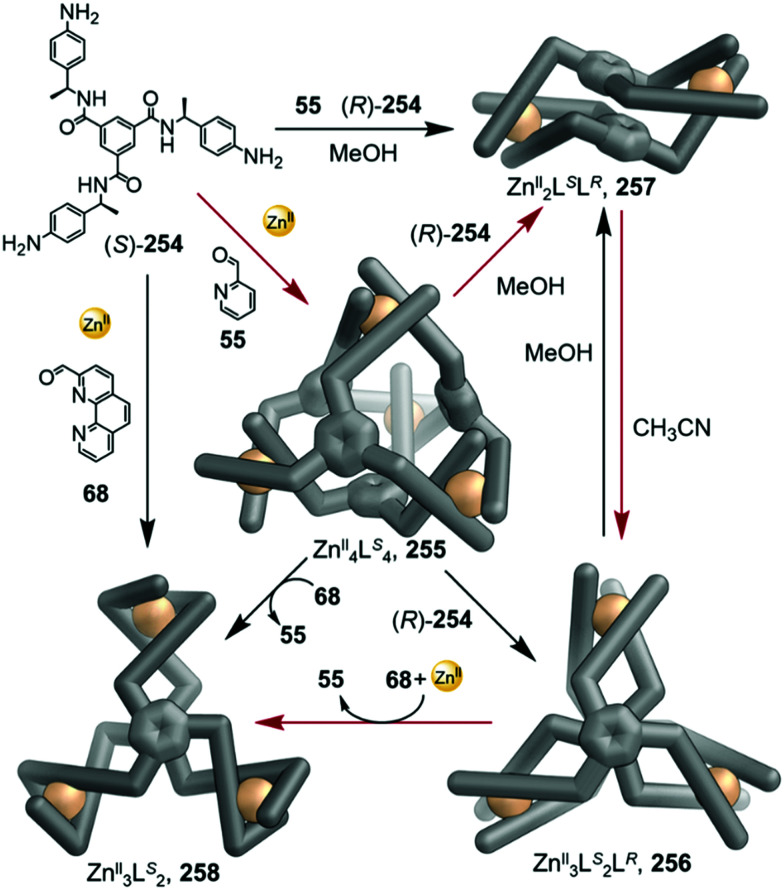
Transformation pathways employing combinations of (*R*)-254, (*S*)-254, 2-formylpyridine 55, 2-formylphenanthroline 68, and Zn^II^. All reactions occurred in MeCN unless otherwise indicated. L^*S*^ and L^*R*^ denote ligands derived from (*S*)-254 and (*R*)-254 respectively. Adapted with permission.^161^ Adapted from ref. 161 with permission from American Chemical Society, copyright 2021.

Fig. 28 shows a complex transformation network, where different combinations of subcomponent exchange and solvent modification drives six cage-to-cage transformations within a system of four different chiral architectures (Fig. 28).^161^ Self-assembly of enantiopure triamine (*S*)-254 with 2-formylpyridine 55 and Zn^II^ in MeOH or MeCN gives the corresponding enantiopure Zn^II^_4_L^*S*^_4_ tetrahedron 255 (where L^*S*^ and L^*R*^ denote ligands derived from (*S*)-254 and (*R*)-254 respectively), having a 3 : 1 *mer* : *fac* configuration with the ligands in an arrangement precluding inter-ligand hydrogen-bonding. Its enantiomer is obtained when (*R*)-254 was employed instead of (*S*)-254. When the two enantiopure tetrahedra are combined in a 1 : 1 ratio in MeCN, a mixture of enantiomers of 256, Zn^II^_3_L^*R*^_2_L^*S*^ and Zn^II^_3_L^*R*^L^*S*^_2_, is formed through a cage fusion process. In each structure, the (*S*)-ligand is stacked between two (*R*)-ligands, or *vice versa*, depending on the enantiomer. This stacked configuration is stabilised by hydrogen-bonding between amide groups. The metal centres within each complex have the same handedness, with *fac* stereochemistry. The intramolecular hydrogen-bonding observed in 256 not only acts as a driving force for the transformation from 255, but also serves to fix the stereochemistry of the final product.

Switching the solvent from MeCN to MeOH induced transformation of 256 into Zn^II^_2_L^*R*^L^*S*^*meso*-structure 257, with two metal centres of *fac* stereochemistry but opposite handedness. Two arms of the same ligand are coordinated to a single metal centre in this achiral assembly, and hydrogen bonds are observed between the enantiomeric ligands. Assembly 257 also forms from 255 following mixture of the tetrahedron with its enantiomer in MeOH. Finally, enantiopure Zn^II^_3_L^*S*^_2_ assembly 258 forms from 255 or 256 by exchange of the bidentate subcomponent 2-formylpyridine 55 for tridentate 2-formylphenanthroline 68. The transformation appears to be driven by the greater number of metal–ligand bonds in the newly-formed architecture.

**Fig. 29:**
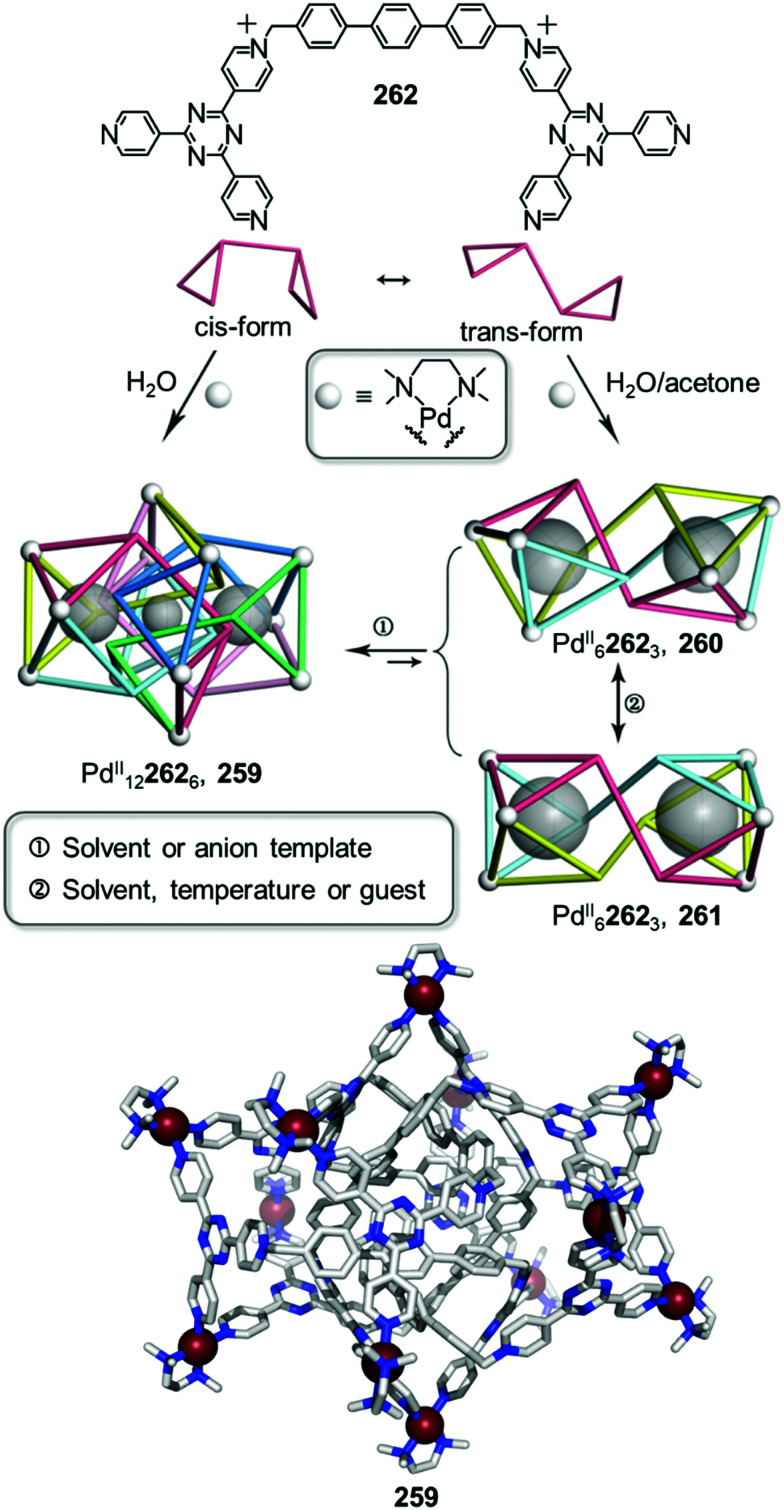
Self-assembly and multi-stimulus-responsive transformations between Pd_12_262_6_ cage 259 and the topologically isomeric Pd_6_262_3_ cages 260 and 261.^162^ The crystal structure of 259 is shown. Adapted with permission. Adapted from ref. 162 with permission from American Chemical Society, copyright 2021.

Solvent also played a critical role in controlling interconversion between Pd^II^_12_262_6_ cage 259 and the two helically isomeric Pd^II^_6_262_3_ cages 260 and 261 in a system described by Sun and co-workers (Fig. 29).^162^ Interlocked *S*_6_-symmetric cage 259 is the sole product observed from self-assembly of the BF_4_^−^ salt of 262 with 2 equiv. of *cis*-protected [(tmen)Pd^II^(NO_3_)_2_] (tmen = tetramethylethylenediamine) in D_2_O, whereas a mixture of the two smaller isomeric cages 260 and 261 is obtained when acetone is introduced during the self-assembly process. The mixture of 260 and 261 converts to 259, indicating that the larger interlocked cage is the final thermodynamic product of the system. The conversion to cage 259 was inferred to be enthalpically favoured by binding of a BF_4_^−^ anion in a central pocket, with a much lower proportion of 259 observed at equilibrium when the NO_3_^−^ salt of 262 was employed.

The semi-rigid 262 ligands adopt a twisted *cis*-conformation in 259, in contrast to the *trans*-configuration in the smaller structures 260 and 261. In *C*_2_-symmetric 260, two ligands interweave and the third ligand does not, while in *D*_3_-symmetric 261 all three ligands are arranged in a helical conformation. The equilibrium between these two cages can be influenced by temperature and solvent, with 261 favoured at higher temperatures, and 260 by higher water content. The threaded arrangement of ligands in 260 reduces its exposed hydrophobic surface area. Adamantane-based guests trigger conversion of 260 to 261*via* an induced-fit guest encapsulation process, with cooperative binding of a total of eight guests between two separate cavities in the structure. This transformation was driven by a better fit of the guests within the larger cavities of 261 (982 Å^3^*vs* 539 Å^3^ for 260). Larger cage 259 also binds adamantyl guests, but with lower affinity, within smaller hydrophobic pockets between the interlocked ligands. Despite weaker guest binding, the thermodynamic stability of the interlocked cage structure was inferred to inhibit structure transformation to 261.

**Fig. 30:**
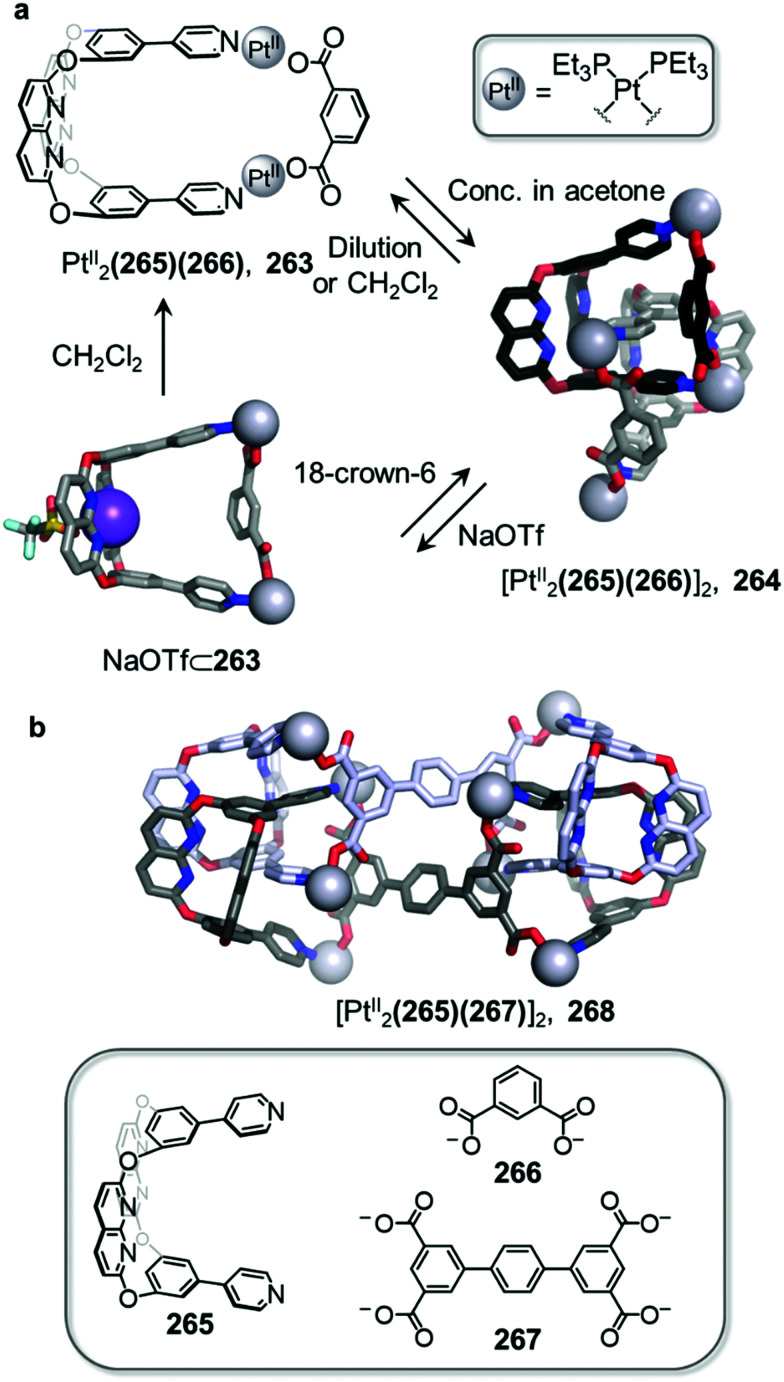
(a) Self-assembly and reversible multi-stimuli responsive transformations between monomeric cage 263 and [2]catenane 264. (b) Crystal structure of cyclic bis[2]catenane cage 268.^163^

A report by Stang, Li, and co-workers demonstrated that changes of solvent, guest-binding, and concentration also resulted in reversible conversion between interlocked and non-interlocked cages (Fig. 30a).^163^ They synthesized heteroleptic Pt^II^_2_(265)(266) cage 263 by self-assembly of *cis*-protected Pt^II^ centres with tweezer-like bis(pyridyl) ligand 265 and bis(carboxylate) ligand 266. The cage was initially isolated as the monomer NaOTf⊂263, with the NaOTf byproduct bound to the naphthyridine spacers of 265. Free 265 was obtained by switching the solvent to CH_2_Cl_2_ and extracting the NaOTf with water. Dimerisation of 263 to form [2]catenane 264, consisting of two interlocked cages, was observed upon crystallisation or in solution when the solvent was switched to acetone and the concentration was increased. The formation of multiple C–H⋯N hydrogen bonds and aromatic stacking interactions between the ligands were the two main driving forces for the stabilization of the [2]catenane 264 over 263.

All transformations in the system (Fig. 30a) are reversible. Monomeric cage 263 reforms upon addition of NaOTf to the cyclic bis[2]catenane 264, and subsequent addition of 18-crown-6 regenerates 264 through extraction of Na^+^, leaving the naphthyridine moieties available to form H-bonds. Addition of CD_2_Cl_2_ to an acetone-*d*_6_ solution of 264 also results in transformation to 263, with CD_2_Cl_2_ proposed to act as a competitive guest in this system, in addition to its role as solvent.

When tetra(carboxylate) ligand 267 is used in place of 266 in the self-assembly reaction with 265, 14-component cyclic bis[2]catenane cage 268 was obtained, with two [2]catenane frameworks interlocked in a similar way as those in 264 (Fig. 30b). Cage 268 showed analogous stimuli-responsive behaviour to 264, transforming into its monomer upon addition of NaOTf, and reconverting into 268 following 18-crown-6 addition. However, 268 was more favoured at lower concentrations than 264 due to an increase in stability attributed to the synergistic effect of the two catenated cages.

**Fig. 31:**
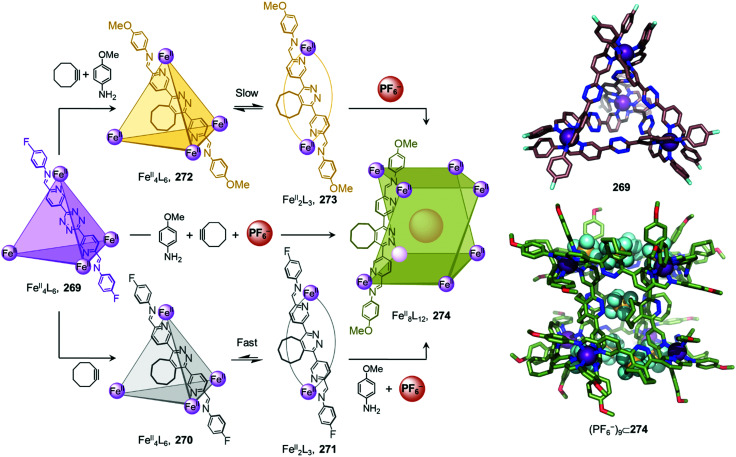
Transformation pathways in a network starting from tetrazine-edged Fe^II^_4_L_6_ tetrahedal cage 269, showing the major products expressed by the system following the addition of different combinations of three stimuli.^164^ The crystal structures of 269 and (PF_6_^−^)_9_⊂274 are shown.

We have explored covalent post-assembly modification (PAM) reactions as stimuli for triggering cage-to-cage transformations.^164^ Many supramolecular PAM reactions proceed with conservation of the original cage framework and are beyond the scope of this review, although we direct readers to other excellent reviews on this topic.^40,41^ As shown in Fig. 31, PAM can introduce instability into a self-assembled architecture in a controlled manner, activating it towards further transformations in response to other stimuli. The reaction of tetrazine-edged Fe^II^_4_L_6_ tetrahedral cage 269 with cyclooctyne *via* an inverse electron-demand Diels–Alder (IEDDA) reaction forms pyridazine-edged tetrahedron 270, which then rearranges to form one of three different architectures after addition of electron-rich anilines or templating anions.

Following PAM, metastable tetrahedron 270 partially converts to the entropically-favoured Fe^II^_2_L_3_ helicate 271, with complete conversion to 271 observed at higher temperatures. The electron-poor 4-fluoroaniline residues of 271 are readily substituted by more electron-rich 4-methoxyaniline, and the resulting tetrahedral cage 272 also undergoes PAM with cyclooctyne, forming an equilibrium mixture of tetrahedron 272 and helicate 273. Interconversion between 272 and 273 is slower than in the previous 4-fluoroaniline-based system, and the equilibrium is shifted in favour of the tetrahedron. Subcomponent exchange also occurs on the mixture of 270 and 271, proceeding more rapidly on the more strained helicate as compared to the tetrahedron.

The application of a third stimulus, PF_6_^−^, to the 270/271 mixture led to a complex mixture of products in solution, including a small amount of Fe^II^_8_L_12_ twisted square-prism 274, which encapsulates nine PF_6_^−^ anions in the solid state *via* stabilizing anion–π interactions. Prismatic structure 274 is the major species observed in solution after addition of the templating anion to the 272/273 mixture, suggesting that all three stimuli are required for its preferential formation. Subcomponent exchange is inferred to have increased the strength of the Fe^II^–N bonds, thus helping to overcome the entropic cost of forming larger Fe^II^_8_L_12_ architecture 274.

None of the structural transformations in this system are possible without first adding cyclooctyne, emphasising the role of PAM as a primary stimulus in this system. The bulky cyclooctyl group is hypothesised to induce the ligands to adopt a nonplanar conformation that promotes formation of the helicate and prismatic architectures. The ability of the three stimuli to bring about structural change in this system thus follows the order PAM > subcomponent exchange > anion templation.

IEDDA reactions have also been used by Jin and co-workers to induce topological transformations between Borromean ring structures and their composite macrocycles in a cascade of transformations that also employs ligand exchange and concentration as stimuli.^165^ More recently the same group has employed the controlled oxidation of thioethers to induce interconversion between Borromean rings and tetranuclear metallacycles,^166^ further demonstrating the potential of post-assembly modification to induce structural transformations of supramolecular architectures.

## 5. Conclusions

This review summarises the diverse strategies which have been used to drive cage-to-cage transformations and create networks of coordination cages by means of one or multiple chemical stimuli. With a better understanding of self-assembly processes, the complexity of these systems has been greatly enhanced over recent years.

Herein, we have highlighted examples in which cage transformations have led to the discovery of unprecedented and often unexpected assemblies, some of which could not be obtained through direct metal–ligand self-assembly. The introduction of competitive or complementary species, such as ligands, subcomponents or metal ions, allows the transformation of one structure into another, and the creation of more complex networks of interconverting structures. These cage-to-cage transformations usually produce the most thermodynamically favourable structure and are thus predictable, providing the thermodynamics of the system are understood. However, reversible processes are challenging to design as the final thermodynamic product cannot be readily transformed back into the original one. In contrast, the use of external stimuli such as templating guests, or changes in pH, solvent or concentration have enabled reversible transformation between cages. Transformations occurring in response to changes in solvent or concentration are particularly advantageous as they do not require additional reagents or generate by-products during the transformation, and are thus cleaner than the other transformation processes discussed herein.

Although many design principles for coordination cages and architectures have been developed throughout the years, it remains challenging to predict their behaviours. The outcome of the combination of rigid ligands and metal ions with well-defined stereo-electronic preferences can often be predicted with a high degree of confidence. However, the effects of ligand flexibility, solvent, concentration, and guest binding are still not perfectly understood. A better understanding of the effects of these stimuli on transformable assemblies may arise from recent advances in machine learning and artificial intelligence. Such efforts to understand the principles behind these transformations and how to predict their outcome will allow the design of more precisely controllable systems for a diverse range of applications.

The current transformations, however are mainly focused upon structural modifications of assemblies, with relatively few examples showing the development of new functions beyond guest uptake and release. The coupling of complex transformations and useful functions thus remains a major challenge for the field. The development of switchable or transformable catalytic systems will greatly benefit from a deeper understanding of transformation processes, and allow chemists to develop more enzyme-like catalysis involving adaptable hosts, targeting new chemical reactions.

Complex signal-driven reconfigurations and cascades represent a way of mimicking biological signalling pathways, where the product from one transformation triggers another, therefore propagating information within the system. Such cascades offer potential routes to controlling the behaviour of complex systems, advancing the development of the discipline of systems chemistry. Such investigations may offer powerful tools to control dissipative^167–169^ or chemically fuelled^170^ systems and create feedback loops. This prospect may also prompt the development of more diverse stimuli, such as light^171^ or electrons,^37^ in order to develop cleaner networks, that respond more quickly to these stimuli than to chemical signals.

Finally, transformable cages could find applications in the field of stimuli-responsive materials. An example might be a system where different functions could be switched on or off as a result of conversion between two functional cages. Reversible transformations are preferable in this context but remain rare because most cage reconfigurations are driven towards a thermodynamic minimum.

The strategies discussed is this review contribute to the growing supramolecular toolbox of methods to transform cages, offering means to create new architectures with useful functions. With the expansion of responsive and stimuli-controlled systems, there is no doubt that this field will continue to flourish in the coming years.

## Conflicts of interest

There are no conflicts to declare.

## Supplementary Material
